# Lymphatic pumping failure in the arm precedes dermal backflow and breast cancer-related lymphedema

**DOI:** 10.1186/s13058-026-02231-w

**Published:** 2026-02-17

**Authors:** Melissa B. Aldrich, Meghan E. McWain, Simona F. Shaitelman, Rian E. Kuhns, John C. Rasmussen, Wendy A. Woodward, Sarah M. DeSnyder, Wenyaw Chan, Eva M. Sevick-Muraca

**Affiliations:** 1 McGovern Medical School, Center for Molecular Imaging, Brown Foundation Institute for Molecular Medicine, University of Texas Health Science Center, Houston, TX USA; 2 Department of Radiation Oncology, MD Anderson Cancer Center, Houston, TX USA; 3 Department of Breast Surgical Oncology, MD Anderson Cancer Center, Houston, TX USA; 4 School of Public Health, University of Texas Health Science Center, Houston, TX USA

**Keywords:** Breast cancer-related lymphedema, Lymphedema, Lymphatics, Near-infrared fluorescence lymphatic imaging, Lymphatic pumping

## Abstract

**Background:**

Breast cancer patients who undergo regional nodal irradiation therapy (RNI) following neoadjuvant chemotherapy and mastectomy or lumpectomy with axillary lymph node dissection (ALND) frequently encounter breast cancer-related lymphedema (BCRL). Early detection and treatment can significantly improve outcomes. Previously, near-infrared fluorescence lymphatic imaging (NIRF-LI) detection of dermal backflow, or retrograde lymphatic flow into initial lymphatics, was shown to precede clinical BCRL diagnosis by 8–23 months. Herein, we hypothesize that lymphatic pump failure is responsible for dermal backflow and may be a target to prevent onset of BCRL symptoms.

**Methods:**

Arm lymphatics in 51 patients with locally advanced breast cancer were visualized with NIRF-LI in a prospective, longitudinal, observational clinical cohort study. Lymphatic pumping frequencies were measured to investigate pumping changes during and after breast cancer treatment. Lymphatic pumping was quantified at five timepoints: after neoadjuvant chemotherapy, but before breast cancer excision and ALND, again at four weeks post-ALND, and at 6, 12, and 18 months post-RNI. Repeated measures mixed-effects analysis was used to compare the mean pumping frequencies amongst all time points. Additionally, pairwise comparisons between any two time points were performed using Wilcoxon matched-pairs signed rank test.

**Results:**

As time lapsed after surgical treatment and RNI, ipsilateral dorsal forearm, ipsilateral ventral forearm, and ventral axilla pumping failure preceded the appearance of dermal backflow and clinically diagnosed BCRL, suggesting that lymphatic pumping dysfunction is the first step in BCRL development.

**Conclusions:**

This study suggests that impairment of the intrinsic lymph pump may be responsible for the onset of clinical BCRL symptoms, and therefore represents a target for prevention of cancer-acquired lymphedema. Future research is needed to understand the molecular determinants of the intrinsic lymphatic pump, and how cancer treatments and interventions can impact them.

**Supplementary Information:**

The online version contains supplementary material available at 10.1186/s13058-026-02231-w.

## Background

Approximately 20–40% of breast cancer survivors encounter incurable breast cancer-related lymphedema (BCRL), a condition of progressive, irreversible arm swelling caused by lymphatic dysfunction [1]. BCRL is clinically diagnosed when the volume of the arm on the cancer-affected side increases by 5–10% over baseline measurements or the volume of the contralateral arm on the untreated side [2]. Cancer treatments of lymph node dissection (LND) and regional nodal irradiation (RNI) are associated with a risk of lymphedema (LE) in breast cancer, but also in other cancers [3–7]. Evidence suggests that BCRL exists in a sub-clinical form prior to irreversible swelling, and that initiating physiotherapy treatments at this stage can stall progression or reverse BCRL symptoms [8–10]. There are no established means to diagnose sub-clinical disease or to assess which cancer survivors will or will not encounter the condition and who could potentially benefit from pre-symptomatic LE treatments. The exact etiology of lymphatic failure that leads to the clinical symptoms of BCRL remains unknown, and there are no strategies to prevent or effectively cure the growing population of cancer survivors with LE.

Recently, we conducted longitudinal near-infrared fluorescence lymphatic imaging (NIRF-LI) studies in advanced breast cancer patients before and after axillary LND (ALND) and RNI. The studies found that the appearance of dermal backflow, or retrograde lymph flow into the initial lymphatic capillaries in the dermis, preceded clinically recognizable arm swelling by 8–23 months [11]. Because dermal backflow occurred distal to the treated site, we hypothesize that failure of the intrinsic lymphatic pump, or the contractile function of lymphangions, causes dermal backflow and the subsequent sequelae of irreversible tissue changes that influence the entire arms of BCRL patients. While others have hypothesized that impairment of the lymph pump is responsible for secondary LE [12–16] and have shown reduced contractile lymphatic activity in the hindlimbs of mice after LND/RNI [17], there has been no direct observation of longitudinal failure of the lymphatic pump in cancer patients who are eventually diagnosed with BCRL. If failure of the lymphatic pump is recognized as an early initiator of cancer-acquired LE, better management strategies to prevent or treat LE could result.

Intrinsic/active and extrinsic/passive factors drive lymphatic pumping. Intrinsic lymphatic pumping depends on phasic contraction/relaxation cycles of smooth muscle cells (pericytes), which surround the largest lymphatic vessels (collectors) [18]. Intrinsic pumping effectors include nitric oxide, histamine, other humoral or neural factors, and lymph pressure/stretch mechanisms [19]. Extrinsic pumping relies on cyclical compression/expansion of vessels by surrounding tissue, such as skeletal muscle, which generates fluid pressure gradients [15].

We hypothesized that sub-clinical etiology of BCRL involves the initial failure of the lymphatic pump that results in distal dermal backflow prior to onset of irreversible swelling. In this study, we measured the lymphatic pumping activity in arm lymphatics prior to the onset of dermal backflow and clinical BCRL diagnosis, to demonstrate the relationship between lymphatic pumping and BCRL in the early sub-clinical stages of disease.

## Methods

### Sex as a biological variable

Only females were included in this study (NCT02949726), because breast cancer primarily affects females. It is unknown whether the findings are relevant for males. Data from a previous study (NCT00833599) was used to establish normal healthy control arm lymphatic pumping frequencies, and these data came from 8 females and 4 males (12 subjects, 24 arms).

### Study subjects and study visit schedule

**Fig. 1:**
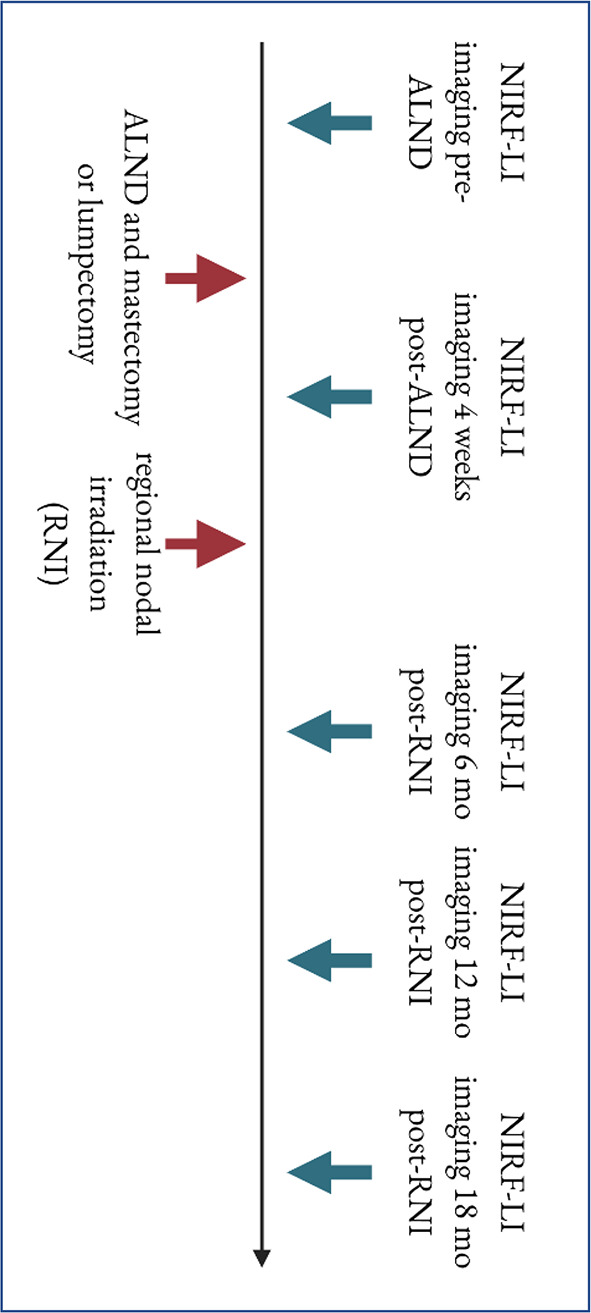

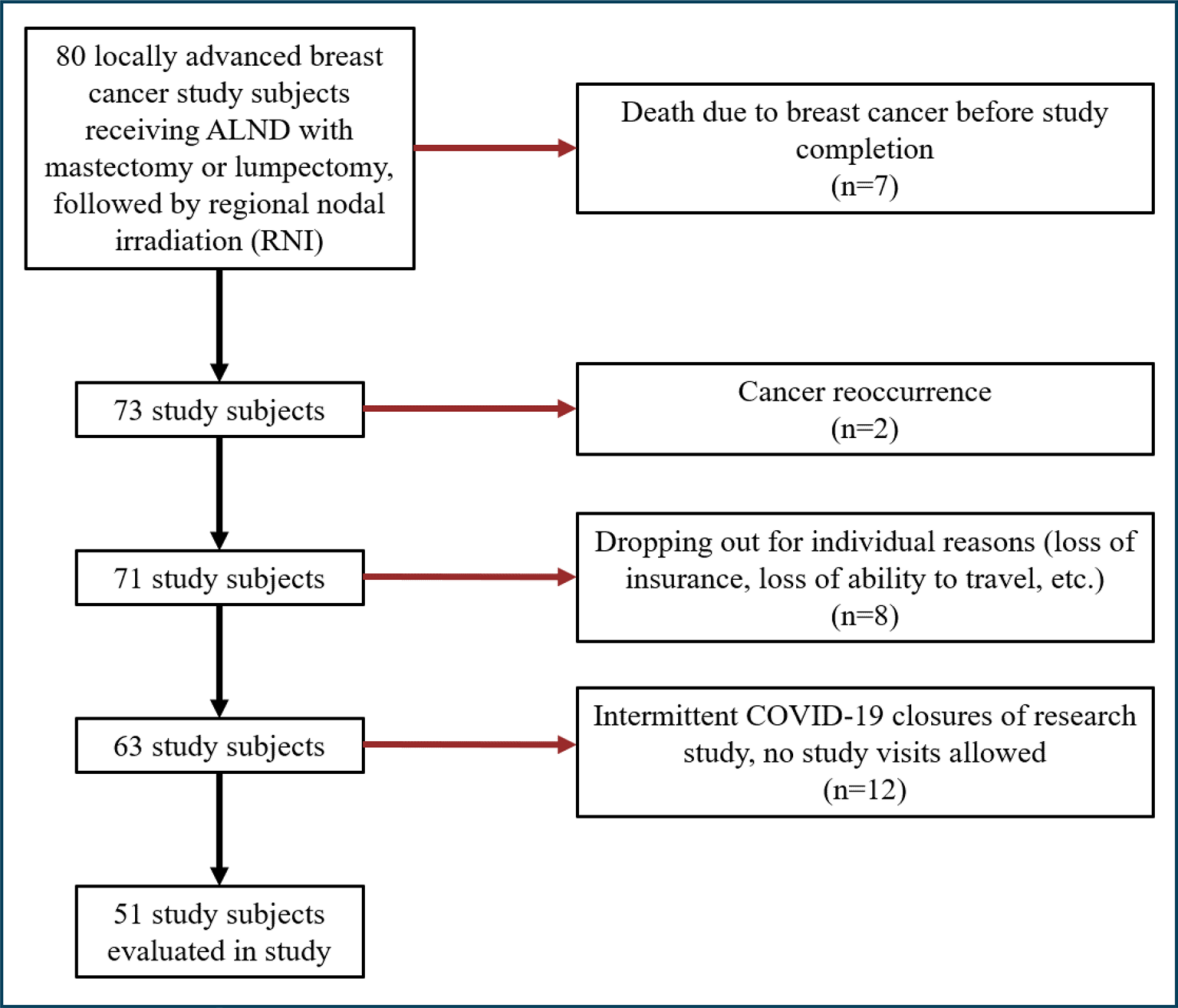
Schematic of study timeline and flow chart for exclusion of subjects lost to death, cancer recurrence. Blue arrows indicate timepoints at which near-infrared fluorescence lymphatic imaging (NIRF-LI) was conducted. ALND=axillary lymph node dissection, RNI=regional nodal irradiation

Fifty-one (51) locally advanced breast cancer patients at MD Anderson Cancer Center were imaged prospectively and longitudinally at baseline, then four weeks after mastectomy or breast-conserving lumpectomy with ALND, and again at 6, 12, and 18 months post-RNI of 60 Gy (Fig. 1). Data collected for NIRF-LI dermal backflow, plasma cytokine/chemokine levels, and patient-reported outcome measures (PROMs) are published elsewhere [11, 20, 21]. Analyses of other protocol-specified data, i.e., immune and genomic data, will come from banked samples yet to be analyzed.

### Study approval

Signed informed consent was obtained from all study subjects, and NIRF-LI was conducted following the principles outlined in the Declaration of Helsinki, with the approval of the University of Texas Health Science Center’s Committee for the Protection of Human Subjects (CPHS) Institutional Review Board (IRB) and the Food and Drug Administration combinational investigation new drug applications (INDs) 106,345 (NCT 02949726, 51 subjects for this study) and 102,827 (NCT00833599, 12 healthy controls in past study) for off-label administration and use of indocyanine green (ICG). All study subject imaging data were de-identified, with hypotheses formulated and variables determined before data analysis (blinded).

### NIRF-LI procedure

NIRF-LI was conducted using a custom-built, variable focus and field of view (350–1900 cm^2^) system, previously described [22]. This system uses a Gen III-intensified charge-coupled device camera and 830 nm bandpass filters to collect fluorescence from indocyanine green (ICG). Skin surfaces were illuminated with < 1.9 mW/cm^2^ 785 nm excitation light, and emitted fluorescence was collected (0.2 s exposure time). At the start of each ~ 70-minute NIRF-LI session, study subjects received 8 intradermal injections of a 0.1 cc, 0.25 mg/mL solution of ICG (Akorn, Inc., HUB Pharmaceuticals, LLC, or Diagnostic Green, LLC). Injections were administered at two sites on the dorsal hand (one medial and one lateral), and two sites on the volar wrist (one medial and one lateral) sites. Four injections were given to the ipsilateral, and four to the contralateral sides. Dorsal forearms, ventral forearms, and medial axillary regions were imaged for approximately 10 min per anatomical area (dorsal aspect of forearm and hand = “dorsal,” ventral aspect of forearm and hand = “ventral,” medial aspect of upper arm and axillary lymph node basin = “axilla”) while the study subjects lay supine.

**Fig. 2:**
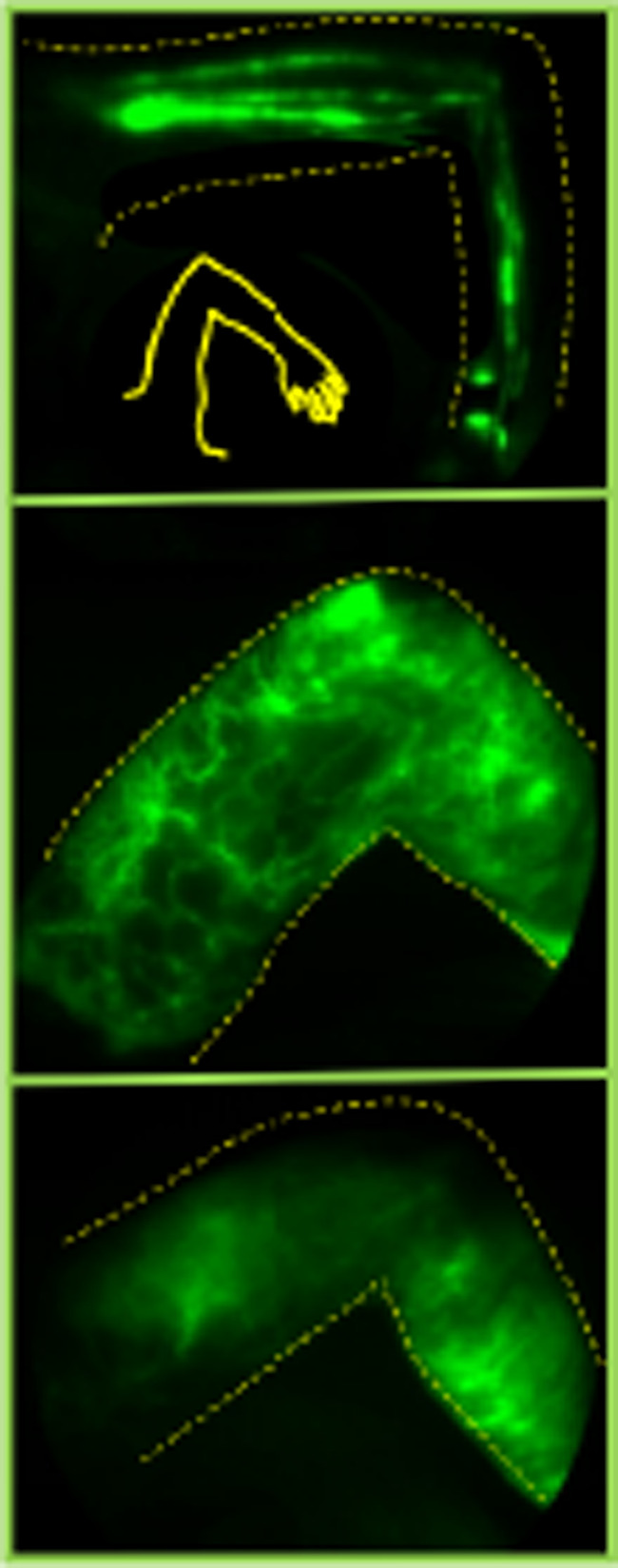
Near-infrared fluorescence lymphatic imaging (NIRF-LI) axilla view of lymphatic collector vessels draining into an axillary lymph node basin in a normal healthy arm (left) and dermal backflow seen in lymphedematous arms (center and right)

NIRF-LI detects lymph nodes and vessels up to 3–4 cm below the skin surface in real-time [20] and from later analysis of image sequences, can provide assessment of lymphatic pumping rates, or contractile frequency. Tiff image files were loaded into ImageJ (NIH, freeware) to generate movies. Regions of interest (ROIs) were selected where fluorescence intensity fluctuations were greatest along visible conducting lymphatic vessels. The number of fluorescence pulses travelling in the proximal direction was divided by the lapsed time (length of the movie, in min) to generate pulses/minute data. Proximal-to-distal (backward) pumping was occasionally observed in the cancer patients, but was counted as zero contractile activity. Figure 2 (left side) shows typical healthy arm lymphatics in an axilla view using NIRF-LI. Figure 2 (center and right side) and Supplemental Video 1 show dermal backflow; retrograde flow or “reflux” potentially reflecting valve dysfunction; vessel dilation that prevents interior valve tip contact and effective retention of lymph within lymphangions; or failure of smooth muscle cells around collector vessels to contract and relax (i.e., the lymphatic intrinsic pump). The diagnostic criterion for BCRL was relative arm volume change (RVC) from baseline of ≥ 5%, measured using a perometer.

When more than one vessel was visible in an anatomical view, vessel pumping frequencies were averaged to produce one value (pulses/minute). Pumping frequencies were plotted at actual values for baseline, post-ALND, and 6 months post-RNI visits. For the 12 and 18 months post-RNI visits, data was omitted for those subject visits at which dermal backflow or clinical BCRL was observed, in order to show pumping frequencies preceding BCRL development. Six of the 51 subjects did not develop clinical BCRL or dermal backflow, so data for those subjects were omitted. Normal pumping frequency values were obtained from 12 healthy control subjects in a previous study, NCT00833599). Pumping frequencies were also reported for all visits as within, below, or above normal pumping frequency ranges.

### Statistics

Repeated measures mixed-effects analysis was used to compare the mean pumping frequencies amongst all time points. Additionally, pairwise comparisons between any two time points were performed using t-test analysis of least-square means (LS-means). Validity statistics such as sensitivity, specificity, and odds ratio were applied to the pumping frequency range data. The Haldane-Anscombe correction (use of 0.5 for 0 in contingency tables) was applied for sensitivity, specificity, odds ratio, and accuracy analysis. Statistical significance was set at *p* < 0.05.

## Results

Table 1 lists demographic data for the 51 NCT02949726 participants. Of the enrolled 80 study subjects, 29 missed more than one of the five scheduled imaging sessions, and were omitted from this analysis. The reasons for missing imaging sessions included death due to breast cancer before finishing the study (7), SARS-CoV-2 interruptions (12), cancer recurrence (2), and dropping out for individual reasons (i.e., loss of insurance coverage, 8). These subjects were excluded because they would not reflect a typical cancer patient with development of BCRL within 18 months post-ALND, and data from those with metastasis/death would confound results. Figure 1B shows a flowchart of subject exclusions.

**Table 1:** Subject demographics (NCT02949726)

Characteristic	Value
Age, median (range)	52 (26–68)
Race, n (%)	
Black	3 (5.9)
Other (Asian, American Indian/Alaska Native, multi-race)	1 (2.0)
White	47 (92.2)
*Ethnicity, n (%)*	
Hispanic or Latino	7 (13.7)
Non-Hispanic	44 (86.3)
*Sex, n (%)*	
Female	51 (100)
Male	0 (0)
Body mass index, mean (range)	29·3 (17.4–46.3)
Underweight (< 18.5), n (%)	1 (2.0)
Normal weight (18.5–24.9), n (%)	10 (19.6)
Overweight (25.0-29.9), n (%)	18 (35.2)
Obese (≥ 30**·**0), n (%)	22 (43.1)
*Clinical T category, n (%)*	
Tx	1 (2.0)
T1	4 (7.8)
T2	20 (39.2)
T3	12 (23.5)
T4b	4 (7.8)
T4d	10 (19.6)
*Clinical N category, n (%)*	
N1	21 (41.2)
N2	4 (7.8)
N3a	13 (25.5)
N3b	1 (2.0)
N3c	12 (23.5)
Neoadjuvant chemotherapy, n (%)	49 (96.1)
Taxanes, n (%)	48 (94.1)
Anthracyclines, n (%)	40 (78.4)
Number of lymph nodes removed at ALND, median (range)	24 (6–42)
Number of lymph nodes involved at ALND, median (range)	1 (0–36)
Lymphovascular space invasion, n (%)	12 (23.5)
Extracapsular extension, n (%)	13 (25.5)
Lumpectomy, n (%)	6 (11.8)
Mastectomy, n (%)	45 (88.2)
Cumulative radiation dose, Gy, median	60
Total number of fractions of radiation, median	30

Of note, 10/51 (19.6%) were diagnosed with inflammatory breast cancer (TNM staging system T4d). All 51 subjects received mastectomy or lumpectomy (11.8% or 88.2%, respectively), with ALND and RNI. Median age was 52 years. Clinically diagnosed BCRL was encountered by 39/51 subjects (76.5%), and dermal backflow was observed in 45/51 subjects (88.2%). Lymphovascular space invasion (LCI) occurred in 23.5%, and extracapsular extension (ECE) occurred in 25.5%. Table 1 shows that 96.1% of subjects received neoadjuvant chemotherapy (the other [not receiving neoadjuvant chemo] 2 subjects both developed BCRL), 94.1% received taxanes (the other [not receiving taxanes] 3 subjects developed BCRL [2] or dermal backflow [1]), and 78.4% received anthracyclines (6 of the 13 non-anthracycline subjects developed BCRL, 8/13 developed dermal backflow). 33.3% received Trastuzumab (7/16 developed BCRL, 10/16 developed dermal backflow). Lower percentages of those taking anthracyclines (46.1%) or Trastuzumab (43.8%) developed BCRL compared to the entire cohort (76.5%). The median number of lymph nodes removed at ALND was 24 (with a median of 1 cancer-involved lymph node). All 51 subjects received a cumulative radiation dose (RNI) of 60 Gy.

**Fig. 3:**
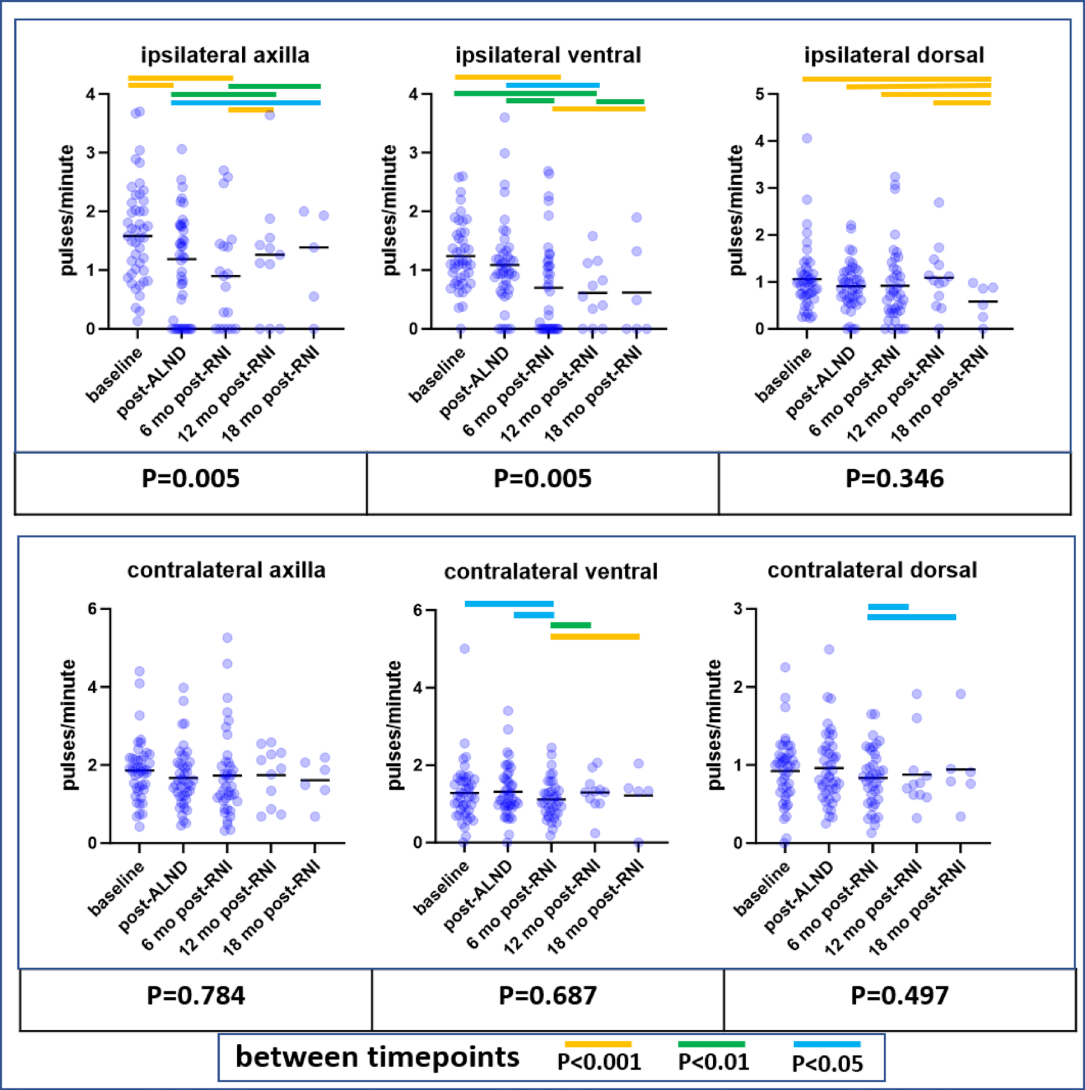
Pumping frequencies for ipsilateral and contralateral medial axilla, ventral forearm, and dorsal forearm imaging views, in pulses/minute. Each point represents one study visit/subject. Values for the 6 study subjects who did not encounter dermal backflow or BCRL are not shown. Horizontal black bars designate mean values.* P*-values for mixed effects analysis, comparing mean pumping frequencies at all time points, are shown underneath each column graph, while p-values for pairwise comparisons (t-test analysis of Least Square Means) between any two time points are shown as colored bars. Orange = *p* < 0.001, Green = *p* < 0.01, Blue = *p* < 0.05

To establish statistical significance of lymphatic pumping frequency changes as BCRL developed, numerical frequency values (pulses/minute, pulses in multiple vessels per view averaged) were plotted for each subject at each visit and are shown in the Supporting Data Values file. Figure 3 shows pumping frequencies and means observed at each study visit for ipsilateral and contralateral dorsal, ventral, and axilla imaging views. At the 12 and 18 months post-RNI visits, data points were excluded for those subjects who already encountered BCRL or dermal backflow, so that only pre-BCRL and pre-backflow data were considered at those time points.

Mean pumping frequencies were significantly different among all time points for ipsilateral axilla and ventral views (*P* = 0.005 and *P* = 0.005, respectively). For ipsilateral dorsal, and all contralateral views, significance was not met (*P* = 0.35, 0.78, 0.69, and 0.50, respectively). For ipsilateral axilla, significant between-timepoint differences in pumping frequencies occurred from baseline to post-ALND, from baseline to 6 months post-RNI, from post-ALND to 12 months post-RNI, from post-ALND to 18 months post-RNI, from 6 months post-RNI to 12 months post-RNI, and from 6 months post-RNI to 18 months post-RNI. For ipsilateral ventral, significant differences in pumping frequencies occurred from baseline to 6 months post-RNI, from baseline to 12 months post-RNI, from post-ALND to 6 months post-RNI, from post-ALD to 12 months post-RNI, from 6 months post-RNI to 18 months post-RNI, and from 12 months post-RNI to 18 months post-RNI. For ipsilateral dorsal, significant differences in pumping frequencies occurred from baseline, post-ALND, 6 months post-RNI, and 12 months post-RNI to 18 months post RNI. For contralateral axilla, no significant differences were noted between timepoints. For contralateral ventral, significant differences in pumping frequencies occurred from baseline to 6 months post-RNI, from post-ALND to 6 months post-RNI, from 6 months to 12 months post-RNI, and from 6 months to 18 months post-RNI. For contralateral dorsal, significant difference in pumping frequencies occurred between post-ALND and 6 months post-RNI, and between post-ALND and 12 months post-RNI. P-values for the between-timepoint mean comparisons are designated with colored bars in Fig. 3.

Supplementary Fig. 1 directly compares pumping frequencies for those subjects who never developed BCRL (with or without dermal backflow) to those with BCRL, without removing data for those subjects with dermal backflow or BCRL. Only ipsilateral axilla frequencies at 12 months post-RNI are significantly different between those who developed BCRL or backflow and those who did not.

Cut-off RVC values to clinically diagnose BCRL can be set at 5% or 10%. For Fig. 3, RVC cut-off was set at *≥* 5%. Supplementary Fig. 2 shows pumping frequencies and p-values for data using *≥* 10% RVC cut-off for BCRL diagnosis. The results show that statistical significance is still met for the ipsilateral axilla and ventral arm views—lymphatic pumping failure precedes the appearance of dermal backflow and BCRL.

**Fig. 4:**
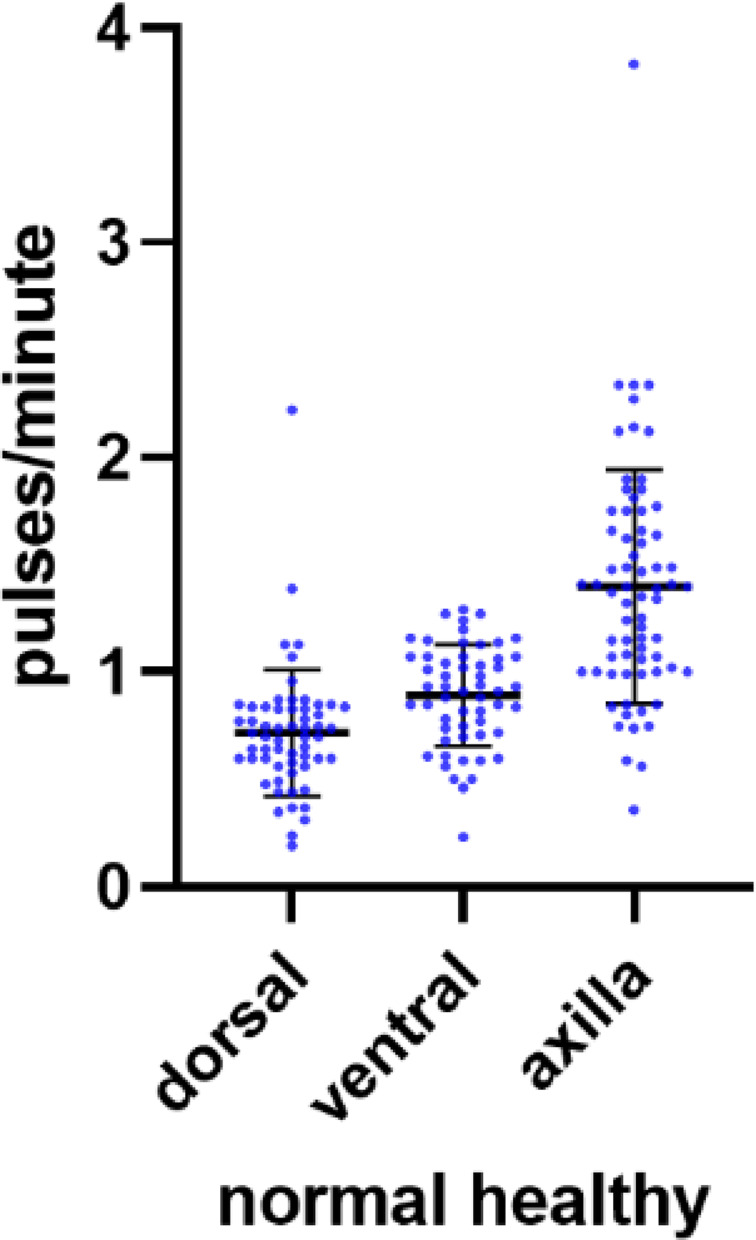
Lymphatic collector vessel pumping frequencies observed in 12 normal/healthy control subjects (24 arms) for arm dorsal, ventral, and axilla views using NIRF-LI, from NCT00833599. Mean values and +/ − 2 standard deviations are shown with horizontal lines

Lymphatic collector vessel pumping frequencies observed with NIRF-LI in 12 normal/healthy subjects (24 arms, from separate study NCT00833599), +/− two SDs, were used to establish normal frequency ranges for arm dorsal, ventral, and axilla views (Fig. 4; Table 2).

**Table 2:** Normal pumping frequencies in healthy/control upper extremities

Location within the upper extremity	Mean pulses/minute (± 2 SDs)
Dorsal	0.79 (0.12–1.32)
Ventral	0.89 (0.41–1.37)
Axilla	1.39 (0.31–2.47)

Supplementary Fig. 3 shows these pumping frequencies separately for male (*N* = 4) and female (*N* = 8) healthy control subjects. Right-side axilla and dorsal view pumping frequencies varied significantly (*P* < 0.01) between males and females—right-side axilla frequencies were higher, and right-side dorsal frequencies were lower, in healthy females. Right-side ventral and left-side axilla, dorsal, and ventral frequencies did not significantly differ.

**Fig. 5:**
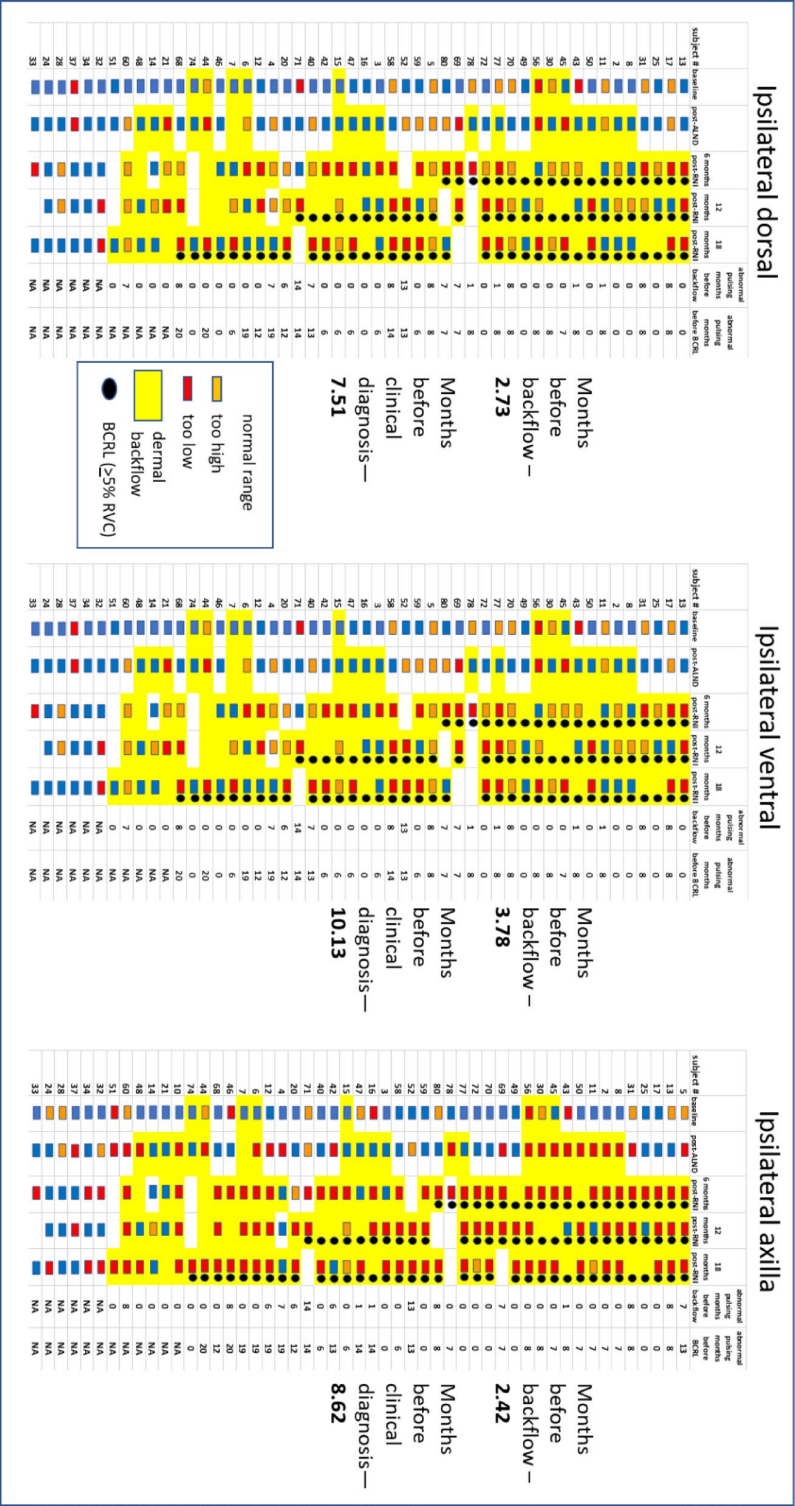

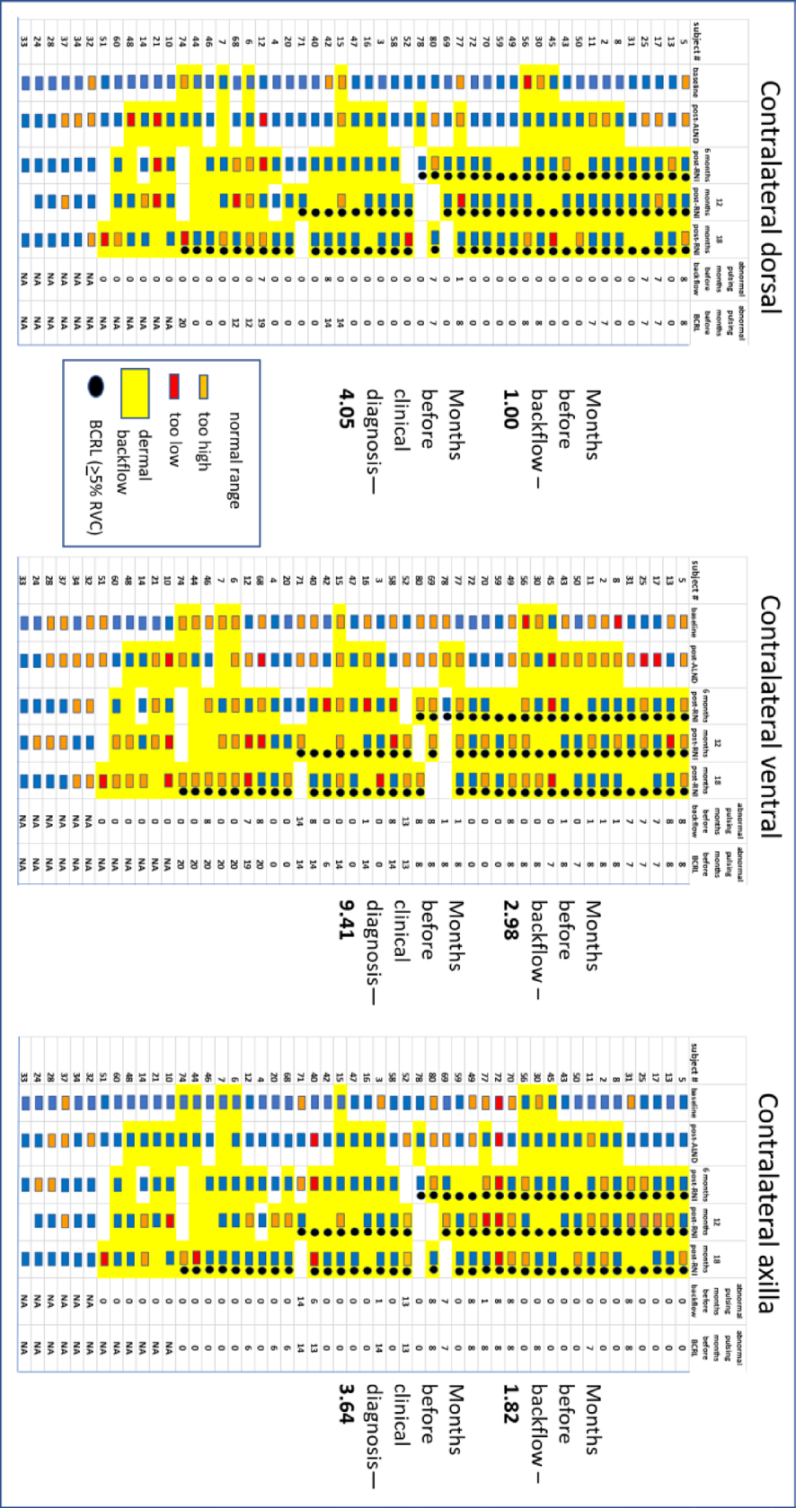
Lymphatic collector vessel pumping frequencies observed in 51 breast cancer subjects for arm dorsal, ventral, and axilla views using NIRF-LI, from NCT02949726. “Below normal,” “within normal,” and “above normal” frequencies are designated by red, blue, and orange rectangles, respectively. Months of appearance of aberrant lymphatic pumping (red/below-normal or orange/above-normal rectangles before dermal backflow (yellow cell background) or clinical BCRL diagnosis (black circle) were averaged for each imaging view. Ipsilateral and contralateral data are shown separately. Relative Volume Change (RVC) cut-off for BCRL diagnosis is *≥* 5%

Pumping frequencies for each cancer subject at each study visit were compiled from NIRF-LI movies, and are depicted in Fig. 5 and categorized as “below normal,” “within normal,” or “above normal” range. The observed frequencies were represented in Fig. 5 as red rectangles for “below normal,” blue rectangles for “within normal,” and orange rectangles for “above normal.” When dermal backflow was observed at any study visit, that chart cell was shaded yellow. When BCRL was clinically diagnosed by *≥* 5% RVC, a black circle was added to the chart cell. Months of appearance of aberrant lymphatic pumping (“below normal” or “above normal”) before backflow was noted, and months before clinical BCRL diagnosis was made, were detailed and averaged for all subjects.

Table 3 shows the average months that aberrant ipsilateral pumping frequencies preceded dermal backflow and clinically diagnosed BCRL (*≥* 5% RVC). Ipsilateral views showed lymphatic pumping dysfunction 2.4–3.8 months before dermal backflow appearance, and 7.1–10.1 months before BCRL appearance.

**Table 3:** Average months that aberrant pumping frequencies preceded dermal backflow and clinically diagnosed (≥ 5% RVC) BCRL

Number of months that aberrant pumping frequency preceded:	Ipsilateral dorsal	Ipsilateral ventral	Ipsilateral axilla
Dermal backflow	2.7	3.8	2.4
BCRL	7.1	10.1	8.6

Table 4 shows high sensitivities for ipsilateral lymphatic pumping dysfunction to predict appearance of dermal backflow and BCRL. Specificities are low or incalculable for all views. Odds ratios for ipsilateral views are above 1.0 for prediction of dermal backflow. Odds ratios for all ipsilateral views are above 1.0 for prediction of BCRL. No odds ratios, however, are statistically significant (*P* values are > 0.05). Accuracies range from 74.5 to 87.5 for dermal backflow prediction, and range from 76 to 77.7 for BCRL prediction.

**Table 4:** BCRL and dermal backflow prediction by lymphatic pumping dysfunction appearance statistics

Dermal backflow prediction	Ipsilateral dorsal	Ipsilateral ventral	Ipsilateral axilla
Sensitivity	85.7	100	97.8
Specificity	22.2	N/A	N/A
Odds ratio	1.7	7.5 *	3.7 *
95% CI	0.3–10.3	0.1-414.2	0.1-121.5
Odds ratio *P* value	0.56	0.34	0.62
Accuracy	74.5	87.5 *	86.4 *
True positives	36	45	44
False positives	7	6	6
False negatives	6	0	1
True negatives	2	0	0

BCRL prediction	Ipsilateral dorsal	Ipsilateral ventral	Ipsilateral axilla
Sensitivity	90	100	100
Specificity	33.3	N/A	N/A
Odds ratio	4.4	3.2 *	10.3 *
95% CI	0.9–21.3	0.1-167.6	0.4-270.4
Odds ratio *P* value	0.06	0.57	0.17
Accuracy	76.5	76 *	77.7 *
True positives	35	39	39
False positives	8	12	11
False negatives	4	0	0
True negatives	4	0	1

*Use 0.5 for zero—Haldane-Anscombe correction

LVI and ECE, both of which increase cancer recurrence risk [23–25], were evaluated using both RVC *≥* 5% and 10% cutoffs. LVI and ECE as drivers of BCRL have not been extensively studied, but show marginally significant association [26]. Supplementary Fig. 4 shows there are significant differences in pumping frequencies for LVI-positive subjects only at baseline for the ipsilateral axilla and ventral views. Significant differences for ECE-positive subjects were seen at baseline for the ipsilateral axilla and ventral views, as well as the 12 months post-RNI ipsilateral dorsal view, only for RVC *≥* 10%.

## Discussion

Failed lymphatic pumping that precedes the development of lymph stasis and observed clinical LE is consistent with the established understanding of the cyclical pathophysiology of secondary LE [27]. Lymphatic injury, inflammation, T lymphocyte or adipose cell dysregulation, and pumping failure could work together to promote clinically observed tissue swelling and fibrosis. The discovery of temporal relationships and molecular determinants of the lymphatic pump will aid in the selection of future optimal therapies.

Of note, pumping loss occasionally appeared as early as baseline (before tumor resection with ALND), perhaps because neoadjuvant chemotherapy or (cancer itself) could influence lymphatic pumping frequencies at baseline. For example, doxorubicin has been shown to interfere with smooth muscle cell contractions and interrupt lymphatic pumping in rats by opening ryanodine receptors [28, 29]. Notably, dantrolene and exercise training can modulate ryanodine receptor 1 dysregulation in human skeletal muscle [30–32], suggesting that the use of a pharmaceutical agent or exercise during neoadjuvant chemotherapy could potentially thwart lymphatic collector smooth muscle destruction or incapacitation. There are several caveats to interpreting pumping frequency in this study. Healthy control data come from a single measurement, and whether these data vary over time is not known. Additionally, baseline/pre-ALND measurements are likely affected by neoadjuvant chemotherapy.

The observed increase in ipsilateral axillary view mean pumping frequencies at 12 and 18 months post-RNI for those subjects who had not yet developed *≥* 5% RVC is inconsistent with the notion that pumping continuously decreases as BCRL develops (Fig. 3). It is unlikely that this increase represents pumping regeneration, as all but 12/51 study subjects ultimately developed BCRL by 18 months post-RNI. Of note, we have occasionally observed higher-than-normal lymphatic pumping frequencies that appear with developing lymphatic dysfunction [33]. We have noticed in mice that, if lymphatic vessels appear to dilate (e.g., in response to an inflammatory insult), the frequency of pumping may initially increase [33]. This increase can associate with a decrease in wave amplitude of a fluorescent bolus of lymph traversing a vessel, due to less vigorous extraluminal lymphatic collector vessel smooth muscle cell (SMC) “peristalsis” of the vessel [33]. Consequently, the frequency of SMC contraction may temporarily increase, but be less intense, and thus less efficient for moving lymph from lymphangion to lymphangion along the collector vessel. This mechanistic explanation is speculative, and additional metrics such as amplitude changes and waveform morphology might provide a more complete explanation.

In this study, almost all subjects received neoadjuvant chemotherapy before mastectomy and ALND and RNI, because the subjects had locally advanced breast cancer. This treatment sequencing may influence BCRL development that differs from BCRL development in patients with upfront surgery treatment sequencing, where patients wait until surgery completion before initiating adjuvant chemotherapy.

While clear trends were difficult to see in Fig. 5, there were more abnormal (too high/too low) pumping values in contralateral compared to ipsilateral views. The ipsilateral axilla, site of ALND, displays more pumping anomalies than other sites, perhaps reflecting surgical disruption of continuous lymphatic vessel anatomy. Abnormal pumping frequencies in those who did not develop BCRL may reflect systemic inflammation that accompanies cancer, ALND, and RNI.

The high rate of BCRL occurrence in our study (39/51, or 76%) was likely due to the study subjects’ locally advanced breast cancer status, with a heavy treatment regimen of neoadjuvant chemotherapy, mastectomy with ALND, and RNI. Notably, 10/51 subjects were diagnosed with inflammatory breast cancer, which elevates BCRL risk [11, 34]. Our recent finding that plasma cytokines/chemokines are significantly elevated before tumor resection/ALND in breast cancer patients who go on to develop BCRL over a year later advocates against surveillance delay until post-RNI or later [20]. In addition, dermal backflow, which was never seen in 24 normal healthy arms imaged in previous study NCT00833599, appeared in NCT02949726 in BCRL 8–23 months before arm swelling *≥* 5%, and never disappeared once present. Thus, there is potential to catch developing BCRL early using tools other than arm swelling, such as NIRF-LI pumping and dermal backflow analysis [11]. As long as arm volume change is used as the primary clinical diagnostic criterion, BCRL detection will be delayed until months after surgery and RNI. Delayed detection misses early windows of opportunity to prevent or stall BCRL development, and to potentially allow for dietary, exercise, or future pharmaceutical prophylaxis.

The use of male and female healthy control study subjects for data shown in Supplementary Fig. 3 as benchmarks may affect the interpretation of whether the all-female BCRL study subjects’ pumping frequencies are below, within, or above normal ranges (Fig. 5). Right-side axilla and dorsal frequencies, which were higher and lower, respectively, in healthy females compared to healthy males, may reflect the small sample size of normal healthy subjects, and could bias analysis. The use of four male healthy controls, together with eight healthy female controls, for measurement of “normal” pulsing frequencies could skew interpretation of comparisons to the BCRL study’s all-female subject composition.

The low specificity results shown in Table 4 indicate that lymphatic pumping is not a good diagnostic indicator. These results suggest that despite good sensitivity, pumping failure as a readout may only hint at a mechanistic explanation for BCRL development, and not serve as a valid clinical screening tool.

Published clinical studies of lymphatic pumping in the context of lymphedema are few, but a cohort of 32 women was imaged at ~ 6 weeks after RNI, and no difference in pumping frequency was found in 6 who eventually developed BCRL after this time point, although contraction velocity was higher in those BCRL-destined [35]. However, one year later in the same cohort, pumping frequencies were reduced in those 6 study subjects [36]. Herein, we longitudinally assess patients across 18 months after cancer treatment to show lymphatic pumping impairment prior to the onset of dermal backflow, which we have previously shown to be a sub-clinical diagnostic of BCRL [11].

Preventative/prophylactic, or immediate lymphovenous bypass (pLVB or iLVB) is growing in popularity, and is becoming the standard of care in some cancer treatment centers. pLVB connects an axillary lymphatic vessel that is severed during tumor resection/ALND to a patent draining vein, immediately after ALND. Some early reports have shown a lower BCRL incidence with pLVB use [37, 38], but at least one study site found no difference in BCRL rates with or without pLVB [39]. As pLVB adoption increases, it may be useful to (1) assess pumping as an outcome facilitator, and (2) consider adding pneumatic compression therapy (PCT) and exercise protocols to recovery regimens in order to restore or preserve pumping function. This approach could benefit patients; a study subject in this study received pLVB at the time of tumor resection/ALND, yet still developed BCRL. NIRF-LI revealed that dye-laden lymph did indeed reach the axillary basins (the pipeline was installed), but the collectors did not pump, and lymph stagnated (Supplementary Fig. 5). Lymphatic collectors that hold but do not pump lymph will not provide sufficient lymph clearance to avoid BCRL-associated detrimental tissue changes [40].

There has been much study of whether ALND is the main driver of BCRL. Our previous work [11] showed that dermal backflow precedes clinically diagnosed (> 5% RVC) BCRL by 8–23 months, and is a hallmark of BCRL and other forms of lymphedema [41]. In addition, dermal backflow was evident in 7 of 45 study subjects before ALND. Another group reported similar observations, with breast cancer study subjects displaying dermal backflow before ALND [42]. The discrepancy between finding dermal backflow before ALND, and the ALLIANCE and other studies that suggest ALND is a major driver of BCRL [43] may be due to (1) breast cancer patients with more severe cancer diagnoses are more likely to be prescribed ALND versus sentinel LND (SLND), and (2) intrinsic injury to the lymphatic pump may occur before disruption caused by surgery/ALND and/or RNI. Interestingly, evidence for LVI and ECE (seen at the time of ALND/mastectomy or lumpectomy) as contributors to lymphatic pumping disruption was only present before ALND.

The limitations of this study include the small sample size and restricted population of breast cancer patients. We surveilled advanced breast cancer patients undergoing ALND and RNI, who are at high risk for BCRL, and are not representative of more common breast cancer treatments experienced by patients with early disease. Another weakness is that all subjects were able to view their NIRF-LI movies in real time, allowing for early warning of subclinical dysfunction. Outside of the study, many subjects then sought out conservative, and sometimes surgical, care for BCRL, before clinical diagnosis. Early compression and/or surgical care/liposuction, by minimizing arm volume, could have also skewed the timing of clinical diagnoses. A total of 26/255 study visits (in the subjects remaining after removing dropouts) were missed due to SARS-CoV-2 outbreaks.

While we collected data for NIRF-LI, plasma cytokine/chemokine levels, and PROMs, analyses for these data are published elsewhere [11, 20, 21], and analyses for immune and genomic data will come from banked samples yet to be analyzed. The previously published analyses uphold the premise that BCRL development involves early, preclinical changes that can be detected with NIRF-LI. In this manuscript, only pumping frequencies are reported and analyzed. Other protocol-specified imaging metrics (vessel dilation and tortuosity) are not reported because initial analyses revealed the parameters were only minimally different in affected and unaffected arms and would not be relevant NIRF-LI imaging parameters for analysis of BCRL severity or onset.

The complete-case approach used in this study may introduce selection bias, especially because attrition was related to death, metastasis, or COVID-19 disruptions. Particularly, because this study enrolled only those patients with locally advanced breast cancer, the results may not apply to those patients with less severe breast cancer, and could make generalization of results invalid for application to the broader breast cancer population. Additionally, the possibility that participants sought early intervention after viewing their own NIRF-LI images may have influenced the timing of clinical BCRL diagnosis.

The planned primary analysis was based on correlations between early arm swelling and multiple imaging and immune parameters. The original power statistics were calculated, based on evaluation of how many study subjects would develop BCRL in a group of 100 locally advanced breast cancer subjects. We assumed a 5% attrition per study visit (23% overall attrition), leaving 77 study-finishing subjects, of whom published results suggested that ~ 30% would develop BCRL. With those assumptions, for two-sided significance or 0.05 and correlation of 0.6 or greater, we would have 85% power. The current analysis, however, focuses on time-based changes in pumping frequency, with a reduced final sample size, so the original power calculations no longer apply, thereby limiting the application of generalizations in interpreting results.

Despite only one significant difference across timepoints between subjects who did or did not develop BCRL (Supplementary Fig. 1), the small sample size underpowers subgroup conclusions.

While over 40% of study subjects had a BMI greater than 30, and subdermal adipose tissue accumulates with higher BMI, NIRF-LI is still able to see shallow lymphatics up to 3–4 centimeters in depth in these subjects [44].

This study’s results suggest that failure of the lymphatic pump in sub-clinical stages may be correlated with future onset of clinical BCRL, and that the causes of lymph pump impairment need to be understood in order to develop more effective, and possibly curative, strategies for the growing population of cancer survivors.

Future BCRL screening could include NIRF-LI or other forms of lymphatic imaging to ascertain collector vessel pumping, inflammatory plasma cytokine screening panels, and the measurement of lymphatic disease biomarkers (such as platelet factor 4, a lymphatic disorder marker) [45]. Assuming that these screening tools, individually or as a panel, are successfully validated, anti-inflammatory dietary or pharmaceutical treatments [46], exercise (an extrinsic lymphatic pump), as well as surgical and/or physical therapy-based early interventions, could be used with conventional LE care to thwart BCRL development at the earliest subclinical stages. New screening tools will require fiscal outlay to develop and validate, but these tools could be considered economical compared to lifelong, troublesome LE care that is not curative and is very costly to medical insurers and patients.

## Conclusions

This study shows that lymphatic pumping disruption can be detected in the affected-side arm before dermal backflow and clinical BCRL appear. This finding points to lymphatic collector pericyte failure as a prominent, and potentially the first, physiological feature of the etiology of BCRL and other forms of LE.

## Supplementary Information

Below is the link to the electronic supplementary material.

Supplementary Material 1.

Supplementary Material 2.

Supplementary Material 3.

Supplementary Material 4.

## Data Availability

Values for all data points used in graphs are reported in the Supporting Data Values file.
