# Supplementary material for: Lymphatic pumping failure in the arm precedes dermal backflow and breast cancer-related lymphedema
Source: Breast Cancer Res. 2026 Feb 17;28:61. doi: 10.1186/s13058-026-02231-w (PMC13014830; doi:10.1186/s13058-026-02231-w)

Supplementary Figure 1. Ipsilateral axillary, dorsal, and ventral pumping frequencies for the 12 subjects who never encountered BCRL (“no BCRL”) and the other 39 subjects who developed BCRL (“BCRL”). *** = P<0.01.

Supplementary Figure 2. Using a 10% RVC cutoff for BCRL diagnosis, pumping frequencies for ipsilateral and contralateral medial axilla, ventral forearm, and dorsal forearm imaging views, in pulses/minute. Each point represents one study visit/subject. Values for the 6 study subjects who did not encounter dermal backflow or BCRL are not shown. Horizontal black bars designate mean values. P-values for mixed effects analysis, comparing mean pumping frequencies at all time points, are shown underneath each column graph, while p-values for pairwise comparisons (t-test analysis of Least Square Means) between any two time points are shown as colored bars. Red=p<0.0001, Orange=p<0.001, Green=p<0.01, Blue=p<0.05.

Supplementary Figure 3. Separate pumping frequencies for male and female healthy control subjects. Green bars designate p-value <0.01.

Supplementary Figure 4. Pumping frequencies for BCRL subjects with or without extracapsular extension (ECE) or lymphovascular invasion (LVI), using >5% or 10% RVC as a cutoff for BCRL diagnosis. (a) 10% RVC, ECE, (b) 5% RVC, ECE, (c) 10% RVC, LVI, (d) 5% RVC, LVI. Green rectangles mark p-values <0.05.

Supplementary Figure 5. A breast cancer subject who received preventive lymphovenous bypass (pLVB) immediately after axillary lymph node dissection. Near-infrared fluorescence lymphatic imaging (NIRF-LI) still images of axilla views at each study visit (pre-surgery/ALND, 4 weeks post-surgery, 6 months post-regional irradiation therapy (RNI), 12 months post-RNI, and 18 months post-RNI. Also shown are pumping frequencies (pulses/minute) for axilla view and arm swelling Relative Volume Change (%RVC) values.

Supplementary Video 1. Backwards flow in an arm, visualized with near-infrared fluorescence lymphatic imaging (NIRF-LI).

Supplementary Figure 1. Ipsilateral pumping frequencies (>5% RVC cutoff), comparing the 12 study subjects who never encountered BCRL to the other 39 subjects who developed BCRL, and comparing the 6 study subjects who never encountered dermal backflow or BCRL to the other 45 subjects.


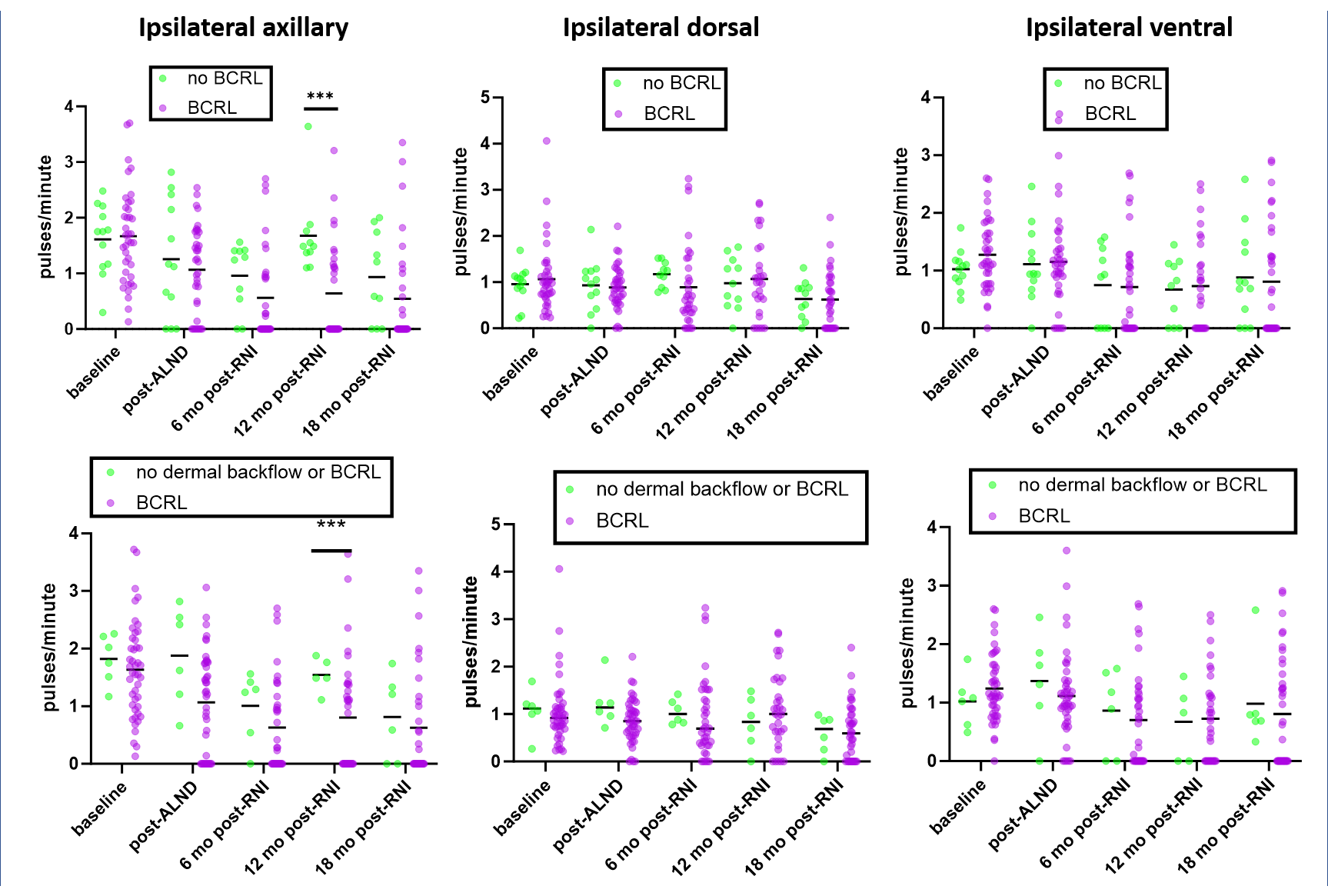


Supplementary Figure 2. Ipsilateral pumping frequencies with 10% RVC cutoff.


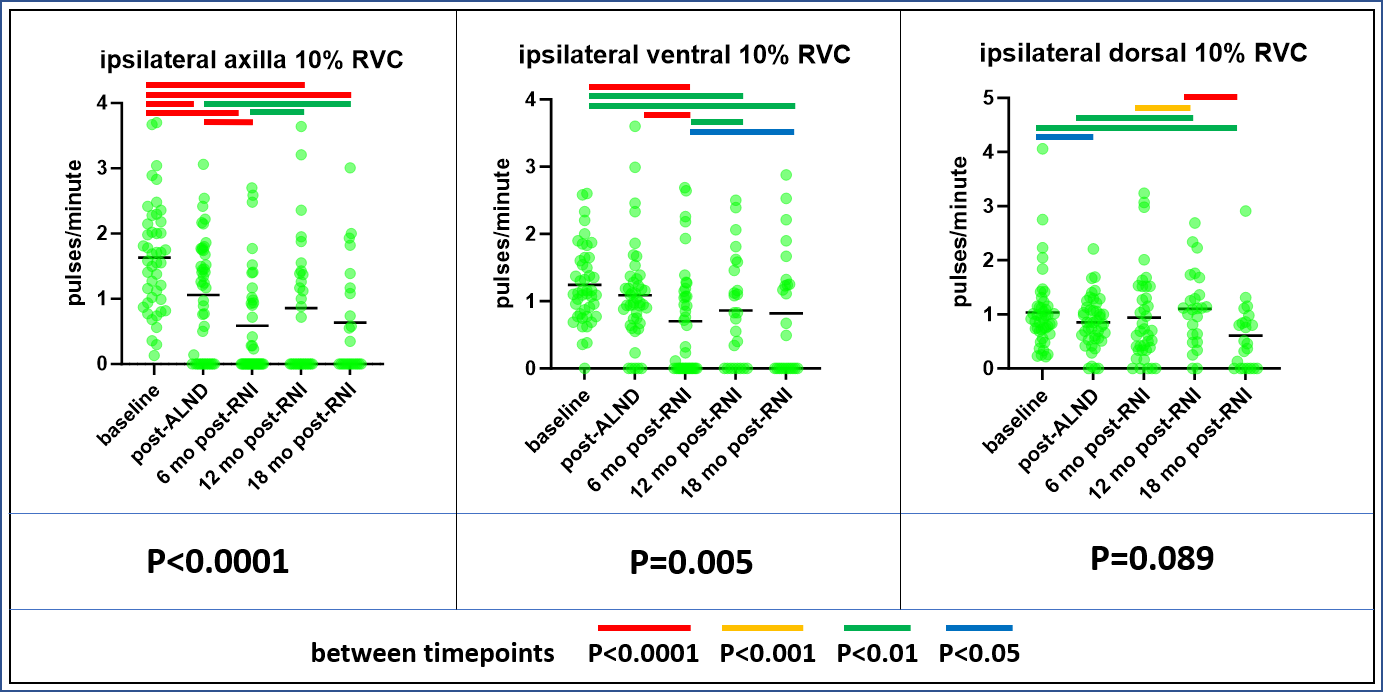


Supplementary Figure 3. Separate pumping frequencies for male and female normal healthy controls.


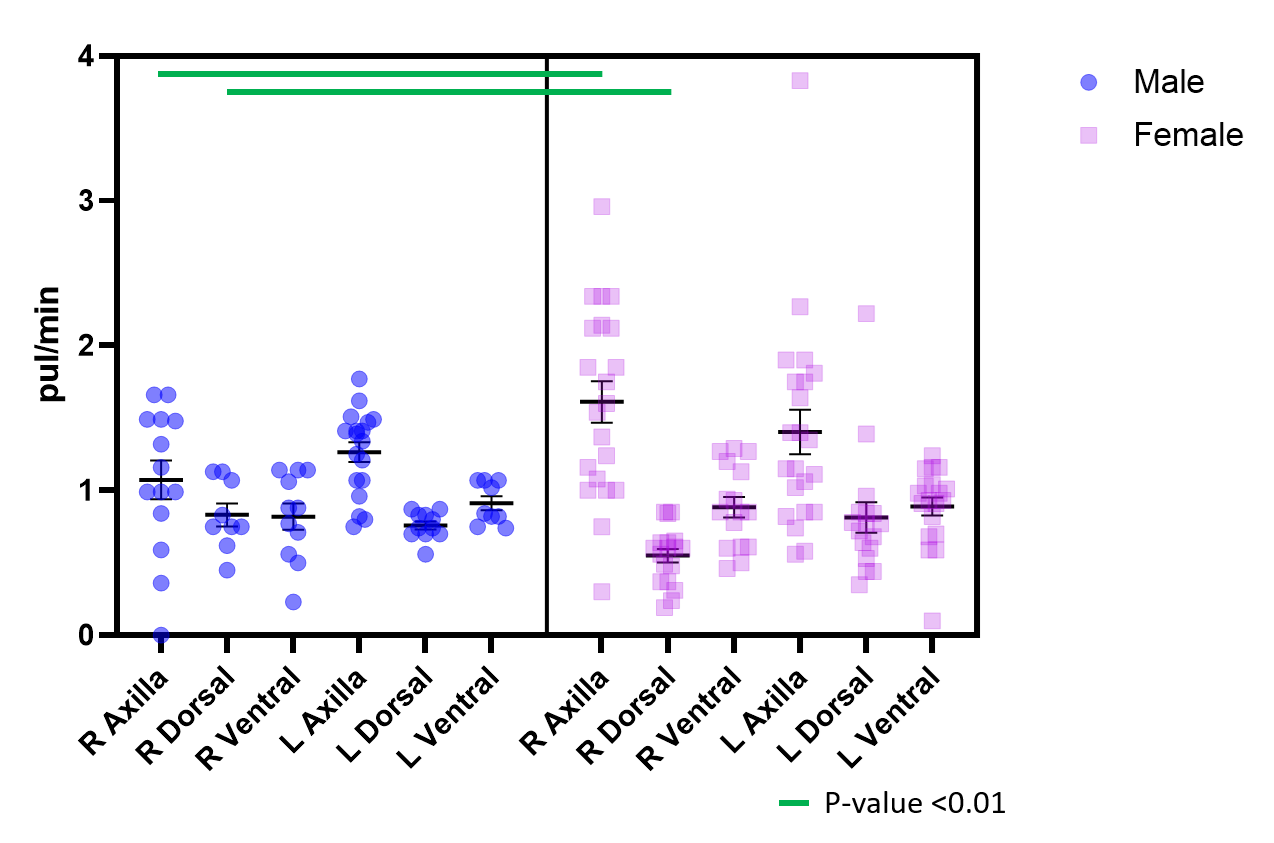


Supplementary Figure 4a. Pumping frequencies using RVC >10% cutoff, extracapsular extension (ECE)


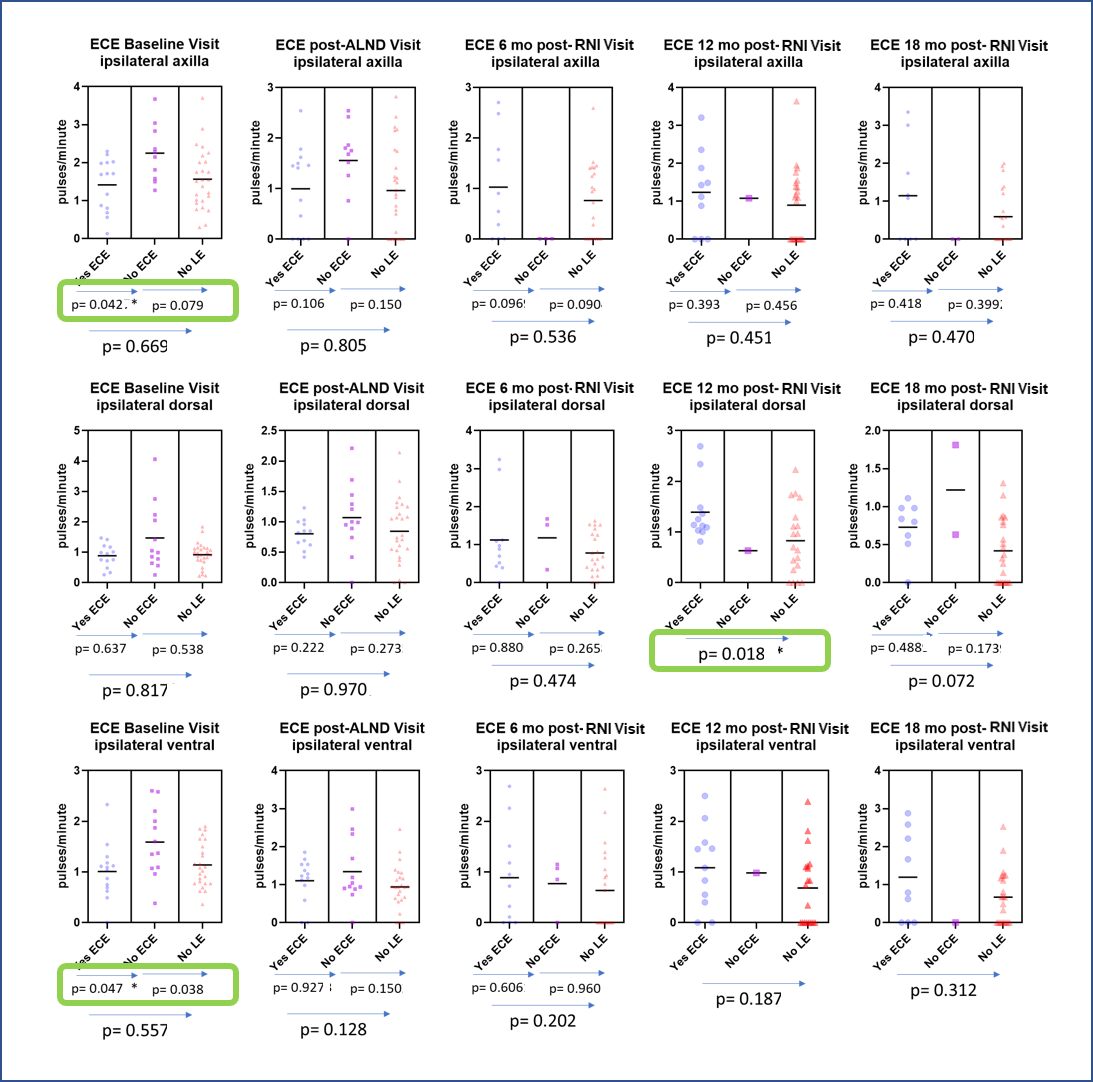


Supplementary Figure 4b. Pumping frequencies using RVC >5% cutoff, extracapsular extension (ECE)


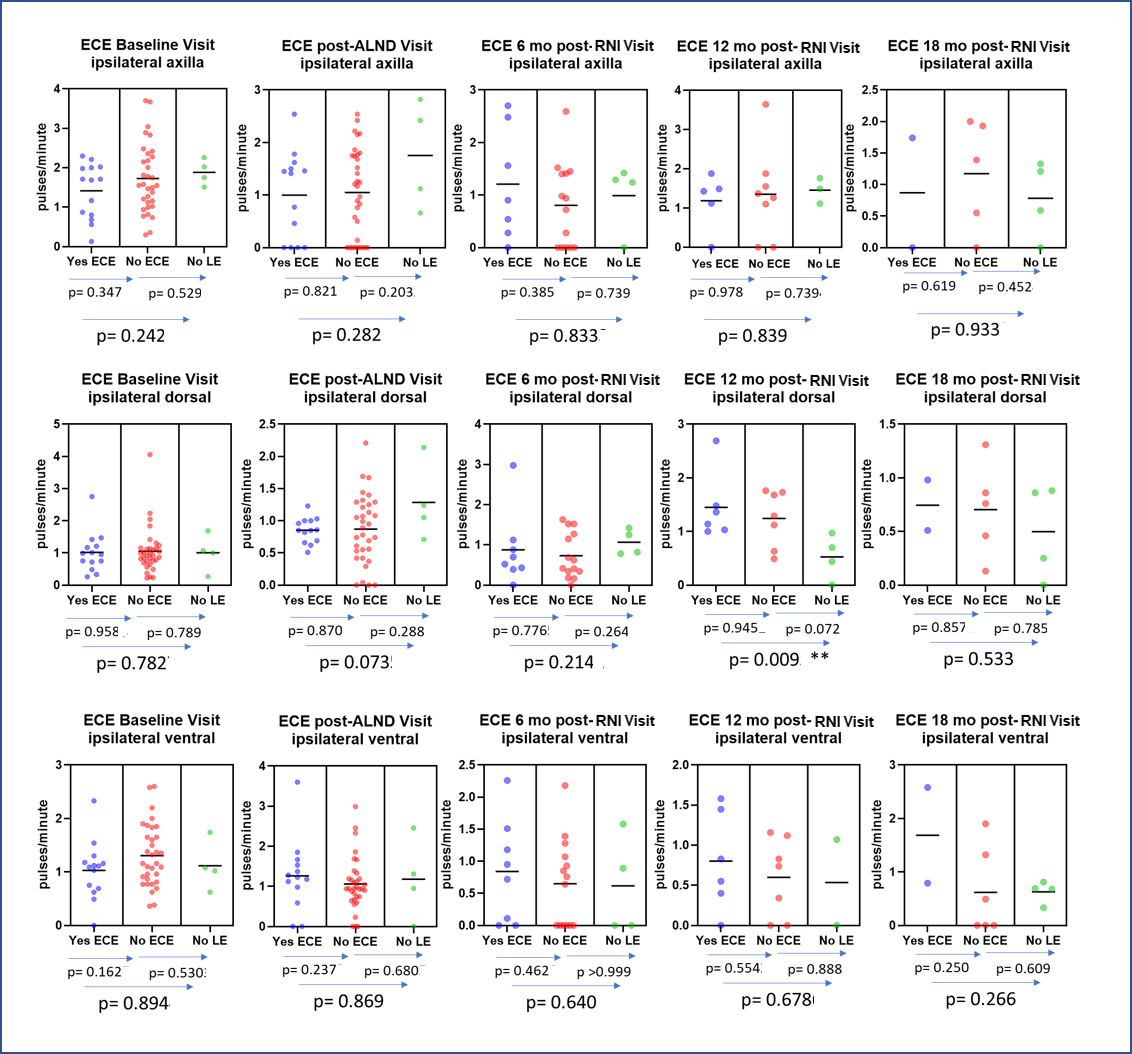


Supplementary Figure 4c. Pumping frequencies using RVC >10% cutoff, lymphovascular invasion (LVI)


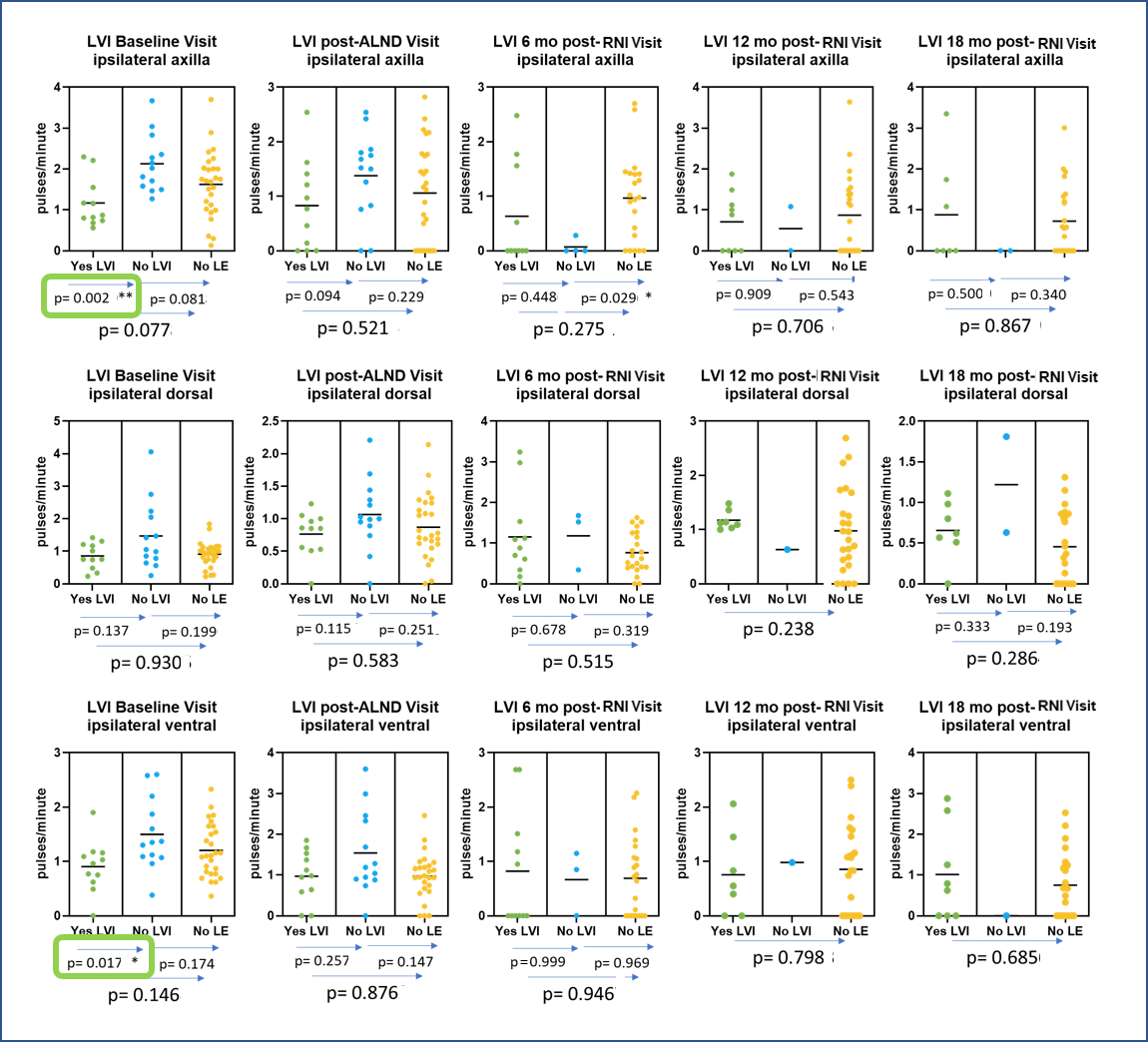


Supplementary Figure 4d. Pumping frequencies using RVC >10% cutoff, lymphovascular invasion (LVI)


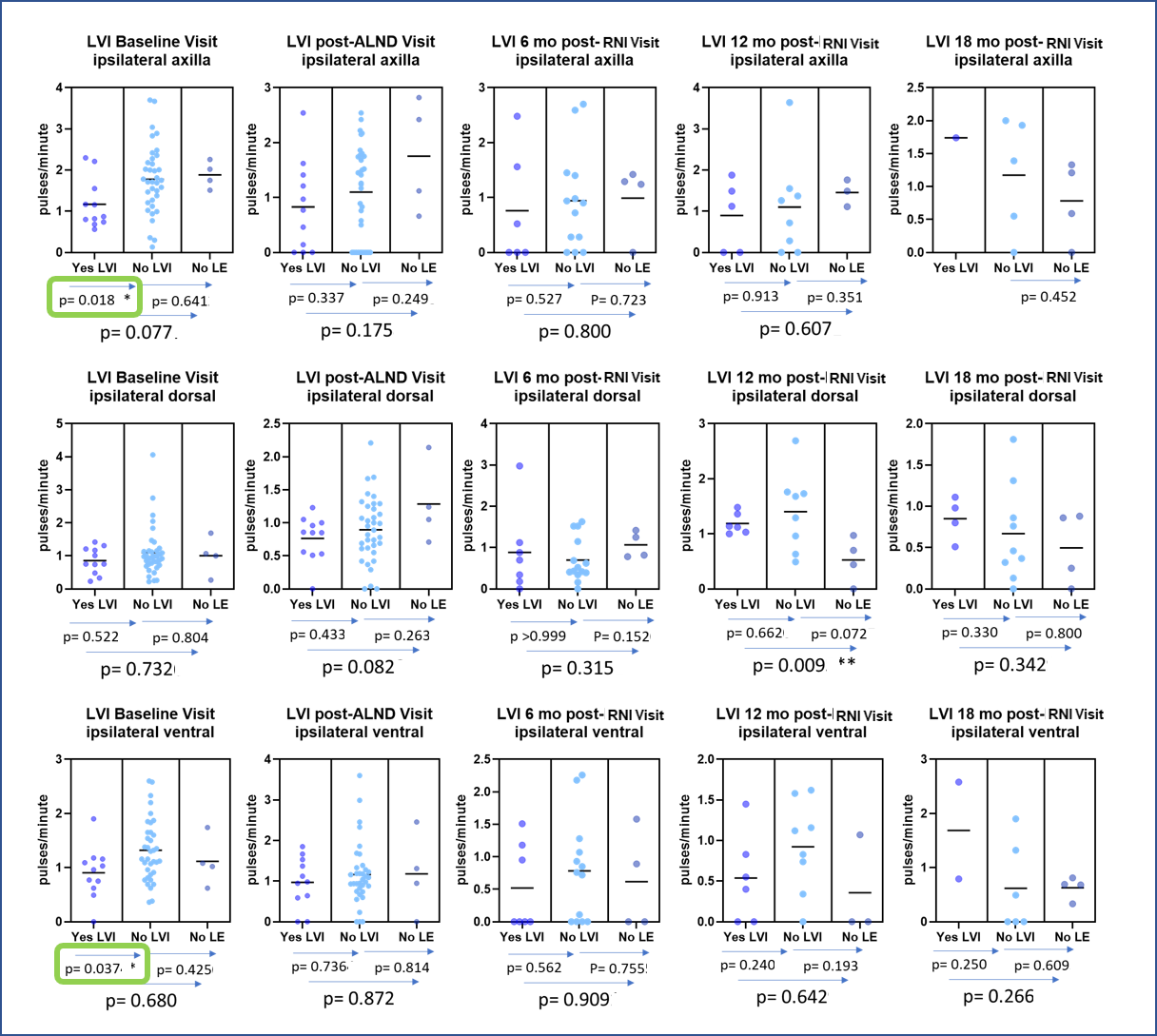


Supplementary Figure 5. A breast cancer subject who received preventive lymphovenous bypass immediately after axillary lymph node dissection.


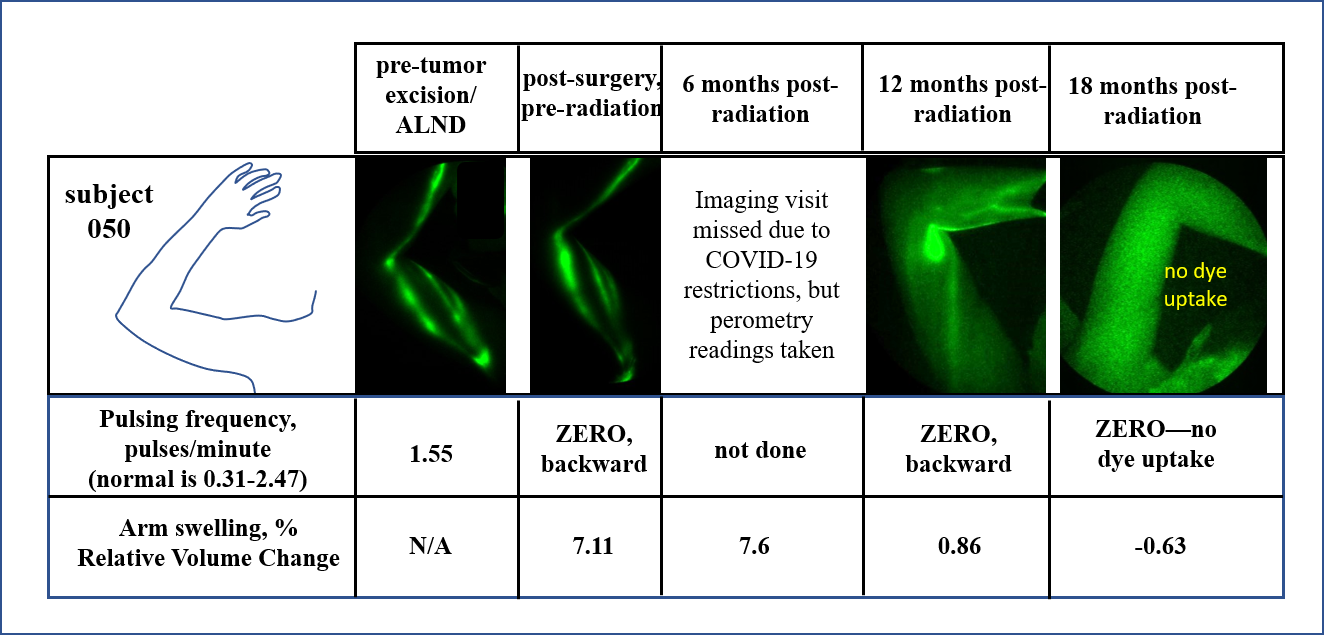

Supplement: Supplementary file 1 — Supplementary Material 1. [file 13058_2026_2231_MOESM1_ESM.docx]
