# Supplementary material for: Lymphatic pumping failure in the arm precedes dermal backflow and breast cancer-related lymphedema
Source: Breast Cancer Res. 2026 Feb 17;28:61. doi: 10.1186/s13058-026-02231-w (PMC13014830; doi:10.1186/s13058-026-02231-w)
Supplement: Supplementary file 2 — Supplementary Material 2. [file 13058_2026_2231_MOESM2_ESM.pptx]

## Slide 1
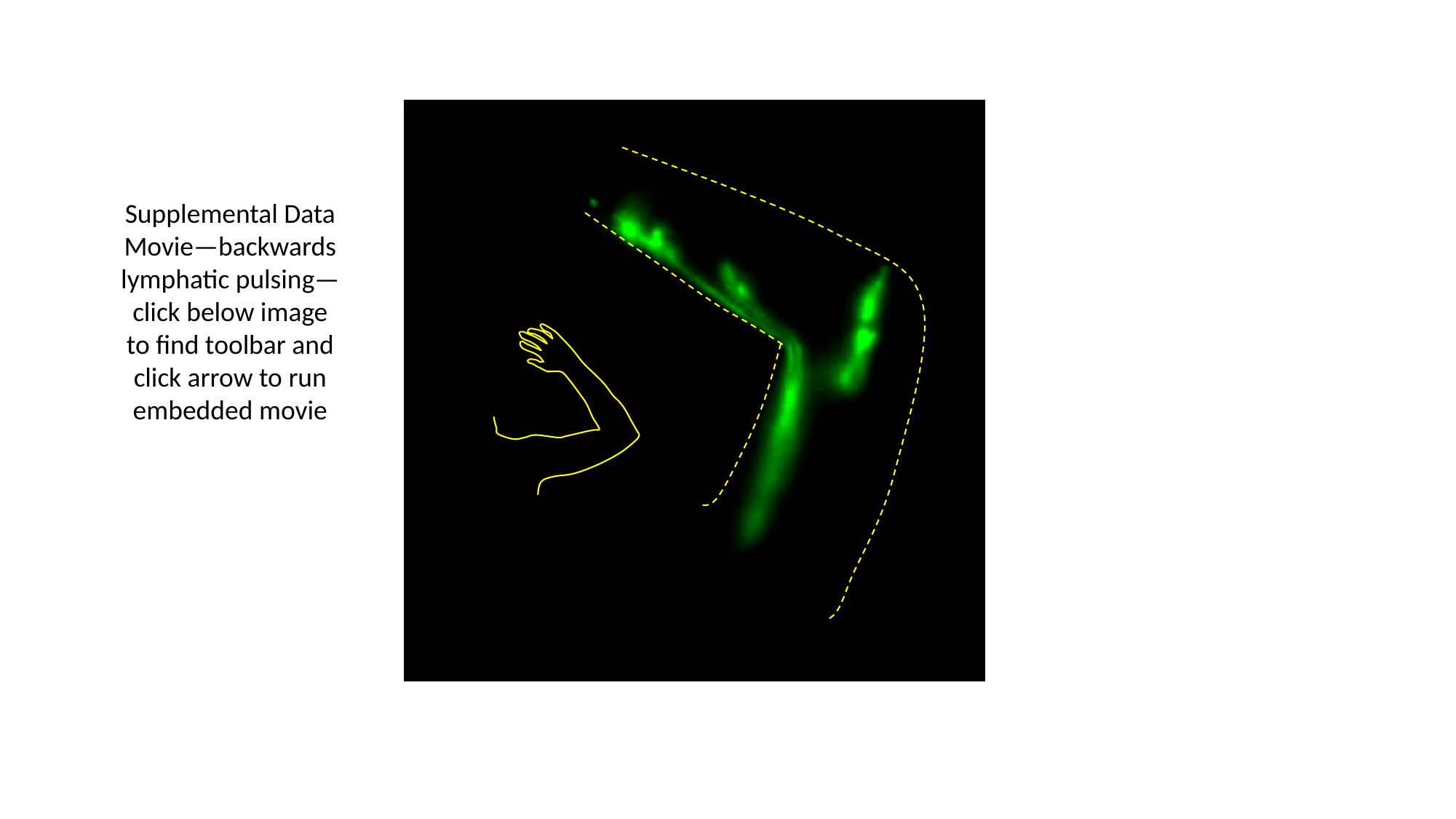

Supplemental Data
Movie—backwards lymphatic pulsing—click below image to find toolbar and click arrow to run embedded movie
