# Supplementary material for: Lymphatic pumping failure in the arm precedes dermal backflow and breast cancer-related lymphedema
Source: Breast Cancer Res. 2026 Feb 17;28:61. doi: 10.1186/s13058-026-02231-w (PMC13014830; doi:10.1186/s13058-026-02231-w)
Supplement: Supplementary file 3 — Supplementary Material 3. [file 13058_2026_2231_MOESM3_ESM.docx]

**Appendix/Supplemental Material**

**Table of contents Page(s)**

1. Study design schematic……………………………………………………………………………... 1
2. Study protocol…………………………………………………………………………………..…..1–13
3. STROBE statement………………………………………………………..………………………13–16
4. **Study design schematic**


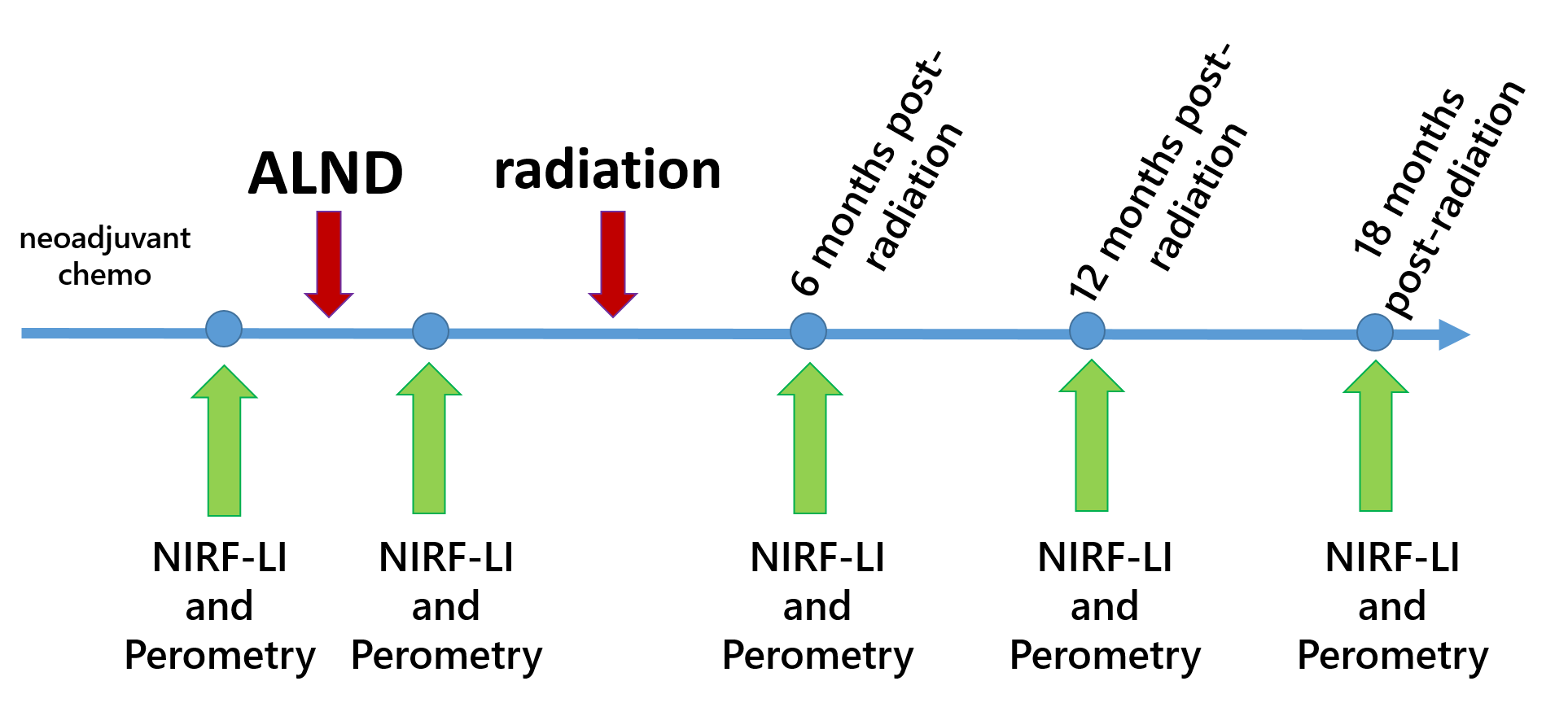


80 advanced breast cancer patients scheduled for mastectomy or breast-conserving surgery with axillary lymph node dissection (ALND), followed by radiation therapy (RT), were surveilled with arm volume measurement/perometry and near-infrared fluorescence lymphatic imaging (NIRF-LI) before and at ~4 weeks after ALND, and at 6, 12, and 18 months post-RT.

1. **Study protocol**

Lymphatic and systemic immunity changes in post-radiation lymphedema

# 1.0 Objectives

This study is conducted in support of NIH Grant 1R01CA201487-01A1, entitled “Lymphatic and systemic immunity changes in post-radiation lymphedema development.” This study intends to prospectively, longitudinally follow breast cancer patients at high risk of developing LE. The study will use Near-Infrared Fluorescence Lymphatic Imaging (NIRFLI) to “visualize” lymphatic vessel anatomy and function, as well as *in vitro* assays to determine changes in serum cytokines and peripheral immune cells. Development of new therapeutics for lymphedema depend on identifying the molecular, cellular, and anatomical changes that occur in the lymphatic system with the onset of swelling, pain, fibrosis, and cellulitis that are the hallmarks of post-cancer treatment lymphedema.

**Primary Aims**: The study will identify markers of the earliest stage of lymphedema by determining correlations between early onset lymphedema (≥ 5% arm volume increase) and:

1. Lymphatic anatomy and function as defined by:
   1. Proportion of extravascular dye: for each still image of a defined anatomical view (for example, dorsal hand), this value will be the surface area covered by extravascular dye, as a percentage of the total surface area of the image. This value will be calculated for each still image (five still images per arm—dorsal hand, medial forearm, dorsal forearm, medial upper arm, dorsal upper arm, axilla).
   2. Lymphatic propulsion rate (frequency and velocity): lymphatic propulsive frequency is normally a value around 1-2 pulses/minute, and lymphatic propulsive velocity is normally a value around 0.8-1.0 centimeters/second. These values are calculated using MetLab software and a software (ALFIA) developed and validated by the investigators (Zhang, J., et al., Biomed Opt Express 2012, 3:1713-23).

c. Lymphatic vessel tortuosity index: the ratio of the actual length of a lymphatic vessel to the cord length of the same vessel, Normally, lymphatic vessels are linear.

d. Lymphatic vessel dilation ratio: the ratio of the width of a lymphatic vessel (measured using ImageJ software from NIH) to the width of the corresponding contralateral vessel.

1. Plasma cytokine and chemokine levels: these values will be expressed in units of picograms/milliliter of plasma, generated using milliplex bead kits analyzed on a Luminex reader. Examination will be performed but not limited to levels of IL-1beta, TNF-alpha, and IL-6.
2. Immune cell function
   1. Plasma antinuclear antibody titers: anti-double-stranded DNA (dsDNA), anti-histone, anti-RNA, and anti-centromere antibodies will be quantified using commercially available ANA testing kits (for example, BioRad EIA autoimmune assay kit). Optical density reading ratios (of patient samples, compared to positive controls provided by kit) will be the values generated. Additionally, we will measure plasma antibodies (using ELISA) to human keratin, collagen, and commercially available skin lysates. Optical density reading ratios (non-lymphedema patient values or pre-lymphedema development values compared to lymphedema patient values or post-lymphedema development values) will be the values generated.
   2. Percent of certain T-cell populations in peripheral blood: these percentages will be generated using uniform gating strategies for peripheral blood T cells that have been stained with antibodies to markers of Th1, Th2, Th9, and other T cell subsets, analyzed with FlowJo software.
   3. Cytokine/chemokine levels in stimulated peripheral blood mononuclear cell cultures: cell culture supernatants, from peripheral blood mononuclear cells that have been stimulated with lipopolysaccharide or toll-like receptor antagonists, will be analyzed for levels of cytokines/chemokines, particularly IL-1beta, TNF-alpha, and IL-6, using milliplex bead antibody kits. Values will be expressed in picograms/milliliter of cell culture supernatant.

**Secondary Aims**:

1) Understanding the correlation of early onset lymphedema with patient-reported outcomes regarding health-related quality of life, satisfaction with outcome, functionality, lymphedema symptoms, and productivity impairment.

2) Obtaining measures of the direct and indirect costs of breast early onset lymphedema will be determined, as will time-driven activity based costs of medical care for those with versus those without early onset lymphedema.

# 2.0 Rationale

Some cancer patients develop lymphedema (LE) after cancer treatment. No one knows exactly who will develop LE or why it occurs.^1^ LE is a chronic disease now affecting 3-4 million people in the United States, and the number encountering LE is expected to rise with the increasing population of cancer survivors.^2^ In LE, the body’s lymphatic system malfunctions, resulting in permanently swollen limbs (and sometimes breasts and trunks) with fibrosis and cellulitis.^3^

Cancer treatment, including surgery, chemotherapy, and radiation, may alter the lymphatic system’s ability to function properly, and 10-40% of cancer survivors in the US develop LE.^4-9^ For reasons unknown, some patients develop lymphedema after cancer treatment, while other patients do not. The patients who do develop lymphedema may encounter swelling immediately after cancer treatment, months after treatment, or sometimes years later.^10^ Research has suggested, but not documented, changes in circulating immune cells and serum cytokines that may drive lymphedema development.^11-1^ In a retrospective study by the co-PI of unilateral breast cancer-related lymphedema (BCRL), lymphatic anatomical abnormalities were found on both ipsilateral and contralateral sides. The incidence of these aberrations increased with time following onset of swelling, suggesting that LE is a progressive, systemic disease.^15^ A more recent study by the co-PI found that key cytokines systemically disrupted lymphatic propulsion. TNF-α (tumor necrosis factor alpha), IL-1beta (interleukin-1 beta), and IL-6 (interleukin-6), were cytokines found to systemically arrest the lymphatic “pump” in mice.^16^ Our evidence showing that LE is a systemic disease, together with increased frequency of cellulitis in LE patients,^2^ suggest that systemic factors, such as cytokines, that are integral to surgical and radiation wound healing throughout the normal inflammation/resolution process, are disrupted or aberrant in LE. Intriguingly, a recent study using a mouse model of vascularized lymph node transplant reported markedly improved healing and anastomoses of lymphatic vessels when a single injection of lipopolysaccharide (LPS) (sterile inflammation) was administered just after surgery, suggesting that alteration or “resetting” of systemic factors/cytokines could improve lymphatic regeneration outcomes after cancer treatment.^17^

This study will be the first to prospectively and longitudinally follow breast cancer patients at high risk of developing lymphedema. Studies by other groups have shown that treating LE in early stages can often prevent development of permanent, more severe disease, and can sometimes reverse disease progression. Currently, there exist only palliative, expensive, time-consuming treatments to control the swelling, and patients are sentenced to a lifetime of compression garment wear, even after new microsurgeries that attempt to create new lymphatic vessel drainage pathways. There is an urgent need to better understand the mechanics and biology of lymphedema. In this study, the near-infrared fluorescence will show changes in lymphatic vessel anatomy/architecture, as well as changes in pumping of lymph from distal arm points to axillary lymph nodes.^18,19^ The investigation of systemic immune changes, particularly plasma cytokines and autoimmune features, will suggest targets for new or repurposed therapeutics for this terrible disease. Additionally, this study may show that near-infrared fluorescence lymphatic imaging can identify the earliest stages of lymphedema. Studies have shown that early lymphedema treatment with standard methods can stall swelling progression.^20^

# 3.0 Eligibility of Subjects

Inclusion criteria:

1) Participants must be at least 18 years of age

2) Participants must be clinically diagnosed with breast cancer
3) Patients must plan to undergo treatment with surgery and radiation therapy at MDACC

4) Clinical stage N2-N3; or clinical stage N1 with an intention to treat with axillary lymph node dissection and regional nodal radiation

5) Ambulatory and possessing all four limbs

6) No prior radiation therapy targeted to lymph nodes

7) Fluency in English or Spanish.

Exclusion Criteria:

1) Participants with a known or suspected allergy to iodine

2) Participants who are breastfeeding, pregnant or trying to become pregnant

3) Severe underlying chronic illness or disease (other than breast cancer)

4) Participants not capable of keeping moderately still for the imaging portion of the study session (~1 hour for imaging)

We will review medical and family history as we analyze data collected at each of the six study visits.


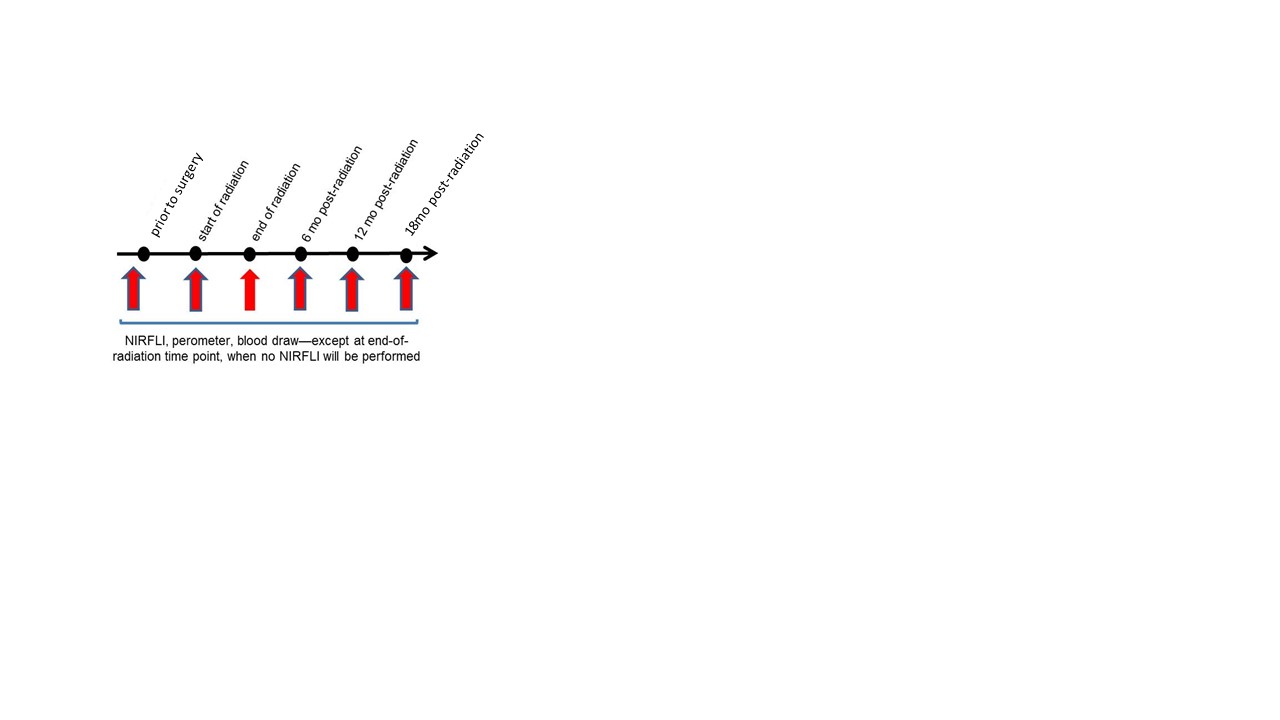


# 4.0 Research Plans and Methods

In order to detect LE development at the earliest stages and provide “before” and “after” comparisons, this study will begin imaging lymphatics before surgery. Additional imaging sessions will occur after surgery but before radiation, and at 6, 12, and 18 months after the conclusion of radiation treatment. No imaging will occur at the end of radiation treatment, due to anticipated patient fatigue. The prospective, longitudinal design of this study will allow correlation of changes in lymphatic anatomy and function, as well as peripheral immune function, with arm, trunk, and/or breast swelling of LE.

## 4.1 Indocyanine Green Imaging Studies

Prior to the start of indocyanine green imaging study sessions, the following two parameters will need to be performed by the patient:

1) Negative urine pregnancy test within 36 hours prior to study drug administration, if of childbearing potential

2) Must complete the Female Pregnancy Evaluation Form(Appendix U). Childbearing potential participants must agree to use one of the medically accepted forms of contraception for a period of one month after study participation. Female participants who meet the criteria for non-childbearing will still complete the Female Pregnancy Evaluation Form, but are not restricted to the use of contraception following study participation

ICG will be prepared as outlined in Appendix II (Preparation of ICG) and Appendix JJ (Standard Operating Procedure for Preparation of ICG Dilution).

Imaging will commence after all injections are made. A diagram of proposed ICG injections sites is shown in Figure 1. Up to 20 injections will be performed into all four quadrants fo the breast and in the subareolar region, as well as into the arm into designated locations. Injections sites will be subject to change, based on factors such as conditions present in limbs or scarring. Multiple sites will be injected to maximize the number of lymph nodes detected by optical imaging.

After injection, near infrared (NIR) imaging will proceed for 30-60 (+/- 5) minutes while the subject is in a supine position. Even though some of the fluorescent light is scattered, we are able to image lymph nodes that are 2-3 cm deep and perhaps deeper. NIR light is generated through low power illumination to create fluorescence of indocyanine green after excitation at 780nm. Documentation of light power is determined before each use. Imaging data are stored in sequential files on encrypted devices that can be played like a video. The acquisition device is a frequency domain photon migration (FDPM) imaging system developed at the University of Texas Health Science Center, Houston. The method of imaging documentation is file stored on an encrypted hard drive.


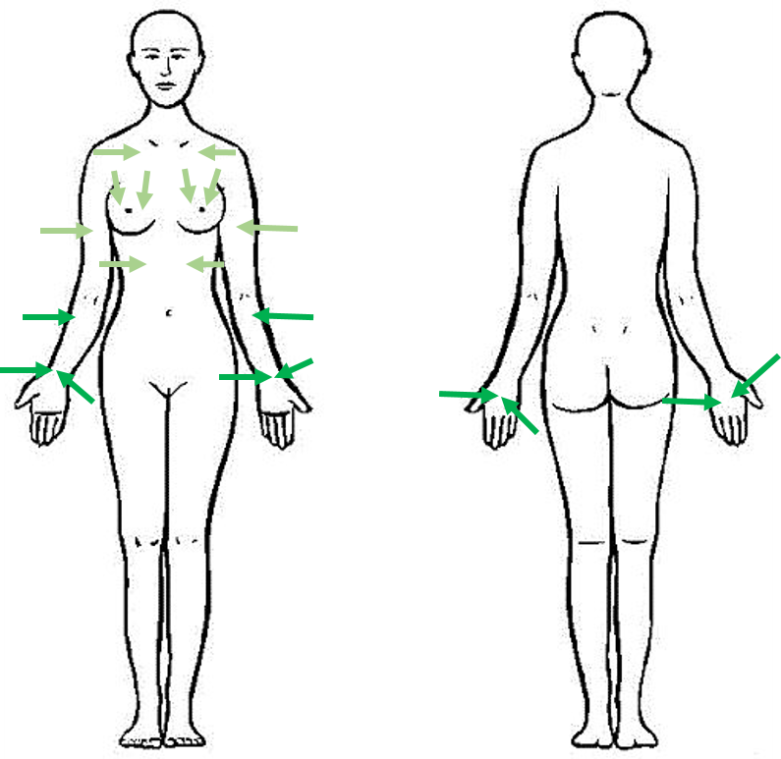


**Figure 1. Proposed ICG Injection Sites**

Lymphatic architecture will also be evaluated. Video images are obtained and the results can be viewed on a monitor. From these files, mapping is performed and compared to known typical lymphatic architecture. We may also observe differences in lymph flow, lymph contractility, or lymphatic architecture that could contribute to the ability of ICG to travel to the axillary lymph nodes. We will evaluate the lymphatic contractility that leads to the propulsive transport of ICG packets (lymph collecting at valves of lymphangions before propulsion) in the lymphatic system, and assess, if possible, the frequency and velocity of packet transit. Briefly, images are reviewed by the Co-PI (Dr. Aldrich) and/or collaborator (Dr. Rasmussen) and areas of interest with lymph trafficking will be identified. Regions of interest are selected from specific lymphatic channels, and the velocity and periodicity of propulsive ICG fluorescence will be measured. This process may be repeated for all other regions in which contractile lymph function is observed, and velocity and frequency averaged. Other parameters, such as lymphatic vessel tortuosity index, vessel dilation ratio, and percent extravascular lymph, will be determined from the videos/images.

Patients will be given a $50 gift card at the time of each ICG imaging study session and for the blood draw conducted at the end of radiation, due to the time and effort involved on their part. A total of up to $300 will be given to each patient for their participation in these studies.

## 4.2 Blood Specimens

At time of each imaging session, as well as at the end of radiation treatment, blood samples (50 mls or less per study visit) will be taken for serum, immune cell, and DNA isolation. We will prepare aliquots of these samples to allow for multiple assays of immune cell and molecule function, as well as future genetic analysis.

Specific variables to be collected/analyzed include but may not be limited to:

1. lymphatic vessels anatomy/architecture
2. lymphatic pulsing frequency (pulses/minute)
3. lymphatic pulsing velocity (centimeters/second)
4. lymphatic vessel tortuosity index
5. percent of extravascular lymph/dye
6. plasma cytokine levels (specifically but not exclusively: TNF-alpha, IL-1 beta, IL-6, IL-12p40, IL-10, IL-13, CXCL10, CCL11, CCL2, IL-8, and IL-7)
7. plasma autoantibody levels
8. plasma levels of antibodies to keratin, collagen, commercial human skin and adipose tissue lysates
9. blood immune cell production levels from *in vitro* assays of peripheral blood mononuclear cells (PBMCs) and granulocytes
10. flow cytometric data (cell type, cytokine production) from PBMCs and neutrophils/granulocytes
11. genomic DNA sequences, particularly of immune-related genes

##

## 4.3 Photography Studies

Photographs will be taken prior to surgery, postoperatively but prior to the start of radiation therapy, and at 6 months +/-2 month, 12 months +/- 2 month and 18 months +/- 2 month following radiation treatment. Photographs will be framed to include the low neck down to the upper abdomen patients will be asked to remove all jewelry prior to photography. Photographs will be taken using a three-dimensional photography suite in the UTMDACC Department of Radiation Oncology. This will be done to enable quantitation of breast/trunk size (volume) and position, and arm volume. All photographic data will be stored in the MD Anderson Box Cloud Storage System and shared by research staff at UTMDACC and UT Health Science Center Houston. Access to the photos will be limited only to those study personnel with a need to view photos. Photographs may also be stored on a password-protected institutional drive with access limited only to those study personnel with a need to view and upload photographs. Photographs obtained in the three-dimensional photography suite will be de-identified through removing the patient’s head from images and, afterwards, such de-identified photographs may be securely transferred to the University of Houston where another study collaborator (Dr. Fatima Merchant) and to University Texas, Austin where another study collaborator (Dr. Mia Markey) is located. Dr. Merchant’s and Dr. Markey’s labs will be able to use sophisticated image processing algorithms to quantitate metrics related to breast size and position. This data will be stored in a secured database system supported by the University of Houston Information Technology Services and University of Texas. Research personnel at University of Houston and University of Texas, Austin will undergo training on research with human participants and on the university's information technology security policies. All data sent to University of Houston and University of Texas, Austin, will be deidentified and coded by patient registration number only. If needed in order to optimize image quality, images will be shared with 3dMD, the manufacturer of the camera used to obtain photographs.

Truncal circumference measurements may be taken using a tape measure at three points: the axilla, 7cm below the axilla, and at the level of the umbilicus.

## 4.4 Optional Cost Analysis

Patients will be offered the opportunity to participate in a detailed cost study, with the goal of capturing up to 50 patients. Costs will include two components: direct medical and indirect costs. The purpose of this is to understand the direct and indirect costs of breast cancer-related care and if this differs in those who do and those who do not develop early onset lymphedema. Direct medical costs will be collected from the time of the completion of radiation up through the finalpost-radiation visit using two sources: institutional databases at MDACC and a patient cost diary. We will apply cost-to-charge ratios to charges recorded in MDACC institutional databases and will obtain counts of various types of healthcare resources (e.g., physical therapy sessions, compressive bandages used to treat lymphedema).

Patients who consent to this additional study will be given a structured, validated cost diary (Appendix T).^23^ Each diary will cover a period of up to 4 weeks and diaries will be given to cover a 6 month time period within the study period following the completion of radiation. Instructions and an example cost diary will be provided to the patient in person (Appendix S). Patients will be asked to record only breast cancer-specific resource use. Patients will record information on the date, practitioner, and frequency of direct healthcare visits (i.e.; visits to physicians, physical therapy, days of hospitalization, prescription medications) and direct non-healthcare costs (ie; over the counter medications, costs of health activities, transportation for medical visits, hours of paid and unpaid household help used), as well as out of pocket costs associated with these. To encourage a high response, prepaid envelopes will be provided to patients to return the booklets. If the diaries are not returned, then patients will be called or electronically contacted and requested to return them as soon as possible. Patient will also be provided with an electronic copy of the diary and if preferable to the patient, these may be returned electronically to research staff. Patients will be provided with a $20 gift card for each 4 week diary that they submit, for a maximum total of $240.

Additionally, we will perform an assessment of the relative difference in time-driven activity based costing for the medical care delivered to breast cancer patients with versus without early onset lymphedema. This determination will be important for healthcare systems to best manage and meet the needs of patients with locally advanced breast cancer who are most likely to develop lymphedema. Time-driven activity based costing will be performed to map the personnel and time resource utilization by breast cancer patients within the healthcare system. The cost per unit time of each provider with whom a patient comes into contact will be calculated, as will the quantity of time for each visit. Process flow charts will be created. These will record medical visits including preoperative evaluation, surgery, postoperative visits, radiation oncology consultation, simulation and treatment planning, radiation delivery, any physical therapy treatments, the first 6 month follow-up visit, and any other interim medical visits at MDACC. Project staff will physically map out these flow charts and track patients for each medical encounter (in person, online, and via phone) conducted with staff at MDACC during the study time period. For each patient, the time resource costs associated with breast cancer treatments and its related follow-up care will be collected. Means of utilization between those patients with and without early onset breast cancer-related lymphedema will be compared.

## 4.5 Patient Assessments

### 4.5.1 Drug Therapy

Patients will or will not have received systemic therapy as per the recommendations of the treating medical oncologist. The following data will be collected (Appendix D, Data Collection Form – Baseline and Appendix E, Data Collection Form--Post Surgery):

- plan for chemotherapy
- timing of chemotherapy (before or after surgery)
- receipt of anthryacycline chemotherapy
- receipt of taxane chemotherapy
- receipt of trastuzumab
- last date of chemotherapy
- names of all chemotherapy agents delivered

### 4.5.2 Surgical Treatment

Patients will receive definitive surgical treatment as deemed appropriate by the surgical oncologist. The following data will be collected regarding surgical treatment (Appendix E, Data Collection Form—Post Surgery)

- final breast surgery performed (segmental mastectomy or mastectomy)
- re-excision performed
- 3-dimensions of gross pathologic specimens removed
- reconstructive surgery performed (yes/no)
- If yes, then type: reduction mastopexy, local tissue rearrangement, placement of tissue expander, implant based reconstruction, TRAM reconstruction, DIEP reconstruction
- Type of lymph node surgery performed (sentinel lymph node biopsy, targeted axillary dissection, axillary lymph node dissection (levels I & II), axillary lymph node dissection (levels I-III), axillary lymph node dissection and internal mammary node dissection, axillary lymph node dissection and supraclavicular lymph node dissection
- Number of lymph nodes removed
- Number of lymph nodes involved with tumor
- Extracapsular extension (yes/no)
- Lymphovascular space invasion (yes/no)
- Date of surgery

### 4.5.3 Radiation Treatment

The treating radiation oncologist will formulate a treatment plan as deemed suitable to treat the patient’s breast cancer. Structures will be contoured and dosimetric data collected as outlined in Appendix G, Dosimetry Data Collection Form.

### 4.5.4 Baseline Data Collection Form

**Purpose:** outlines protocol-specific baseline characteristics and includes the following (Appendix D-Data Collection Baseline Form):

- Patient age
- Race (white, black, Asian/pacific islander, other)
- Origin (Hispanic/ Non-Hispanic)
- Menopausal status (pre-menopausal, peri-menopausal, post-menopausal)
- Weight (in kg)
- Height (in centimeters)
- Personal history of lymphedema (yes/no)
- Family history of lymphedema (yes/no)
- Personal history of autoimmune disorder (yes/no). If yes then explanation of type
- Frequency of exercise (number of days per week participate in 20 minutes or more of exercise)
- Type of exercise (aerobic, anaerobic)
- Tobacco use (yes/no)
- Clinical T stage
- Clinical N Stage
- Quadrant of breast tumor
- Side of breast tumor (right, left, or bilateral)
- Estrogen receptor status
- Progesterone receptor status
- Her2-neu status
- Plan for type of breast surgery (segmental mastectomy, mastectomy with no reconstruction, mastectomy with reconstruction)

**Timeline for completion:** at time of registration

### 4.5.5 Arm volume calculations:

**Purpose**: to provide a validated measure of arm volume over time

Perometer measurements will be taken of the arm at the following time points: (see Appendix C-Study Parameters): prior to surgery, prior to radiation, 6 months +/- 2 month, 12 months +/- 2 month, and 18 months +/- 2 month following completion of radiation.

Perometer measurements will include:

- Bilateral arm volume (in ml) (three measures of each arm)
- Length of arm measured (in cm)

Research staff will follow the Perometer Standard Operating Procedure (Appendix B).

### 4.5.6 Breast/truncal photographs:

**Purpose:** to create a photographic record of the appearance of the patient’s breasts/chest wall, to be used as a part of lymphedema assessments.

- 3D photographs and truncal circumference will be obtained.

**Timeline for completion:** completed prior to surgery, prior to the initiation of radiation, 6 months +/- 2 month, 12 months +/- 2 month, and 18 months +/- 2 month following completion of radiation.

### 4.5.7 Post-Surgery Data Collection Form

**Purpose**: outlines protocol-specific cancer related parameters determined after surgery and includes the following (Appendix E-Data Collection Form Post Surgery):

- Chemotherapy:
  - Neoadjuvant chemotherapy (yes/no)
  - Anthracycline chemotherapy (yes/no)
  - Taxane chemotherapy (yes/no)
  - Trastuzumab (yes/no)
  - Chemotherapy agents
  - Last date of chemotherapy
- Pathologic T stage
- Pathologic N stage
- Gross dimensions of specimen removed at surgery (cm)
- Type of lymph node surgery performed (sentinel lymph node biopsy, targeted axillary dissection, axillary lymph node dissection (levels I & II), axillary lymph node dissection (levels I-III), axillary lymph node dissection and internal mammary node dissection, axillary lymph node dissection and supraclavicular lymph node dissection
- Number of lymph nodes removed
- Number of involved lymph nodes
- Extracapsular extension (yes/no)
- Lymphovascular space invasion (yes/no)
- Final breast surgery performed (segmental mastectomy/mastectomy)
- Date of oncologic breast surgery
- Reconstructive surgery performed (yes/no)
  - If yes, then type: reduction mastopexy, local tissue rearrangement, placement of tissue expander, implant based reconstruction, TRAM reconstruction, DIEP reconstruction
- Re-excision performed (yes/no)
  - If yes then 3 dimensions of pathologic specimen

**Timeline for completion:** after surgery, before start of radiation treatment

### 4.5.8 Follow Up Data Collection Form

**Purpose**: outlines protocol-specific treatment-related factors and includes the following (Appendix H (Data Collection Follow Up Form):

- Weight
- Frequency of exercise
- Type of exercise done
- Usage of endocrine therapy
- Type of endocrine therapy
- Usage of compressive sleeve garment for lymphedema
- Date and type of the following procedures if done:
  - breast cosmetic procedure (ipsilateral or contralateral): lift, reduction, fat transfer, other;
  - breast oncologic procedure (ipsilateral or contralateral): lumpectomy, mastectomy, other;
  - reconstructive surgery (ipsilateral or contralateral): TRAM reconstruction, DIEP reconstruction, latissimus dorsi flap reconstruction, implant based reconstruction, other
- Healthcare provider assessment of patient
  - Arm edema
  - Breast/chestwall edema
  - Breast/chestwall erythema
  - Breast/chestwall thickening
  - Peau d’orange of breast/chestwall

**Timeline for completion**:6 months +/- 2 month, 12 months +/- 2 month, and 18 months +/- 2 month following completion of radiation.

### 4.5.9 Quick DASH (Disabilities of the Arm, Shoulder, and Hand) (Appendices J, K)

**Purpose**: Evaluates patient symptoms and functionality of the upper extremity. This form is available in both English (Appendix U) and Spanish (Appendix V) and patients should be allowed to use the language that they prefer.

**Timeline for completion**: prior to surgery, prior to radiation therapy, 6 months +/- 2 month, 12 months +/- 2 month, and 18 months +/- 2 month following completion of radiation.

### 4.5.10. Lymphedema Symptom Intensity and Distress Survey-Arm (Appendix I)

**Purpose**: This form captures information on patient symptoms associated with arm lymphedema. The questionnaire addresses six clusters of symptoms: swelling, pain, movement, mood/confidence, insurance and sex. This form is available in English only. Patients fluent only in Spanish are not required to fill it out.

**Timeline for completion**: prior to surgery, prior to radiation therapy, 6 months +/- 2 month, 12 months +/- 2 month, and 18 months +/- 2 month following completion of radiation.

### 4.5.11. Patient Lymphedema Symptom Survey Instrument (Appendix M)

**Purpose**: This form captures information on patient symptoms of breast edema. This form is available in English only. Patients fluent only in Spanish are not required to fill it out.

**Timeline for completion**: prior to surgery, prior to radiation therapy, 6 months +/- 2 month, 12 months +/- 2 month, and 18 months +/- 2 month following completion of radiation.

### 4.5.12 Work Productivity and Activity Impairment Questionnaire: Specific Health Problem (v2.0) (Appendices N, O)

**Purpose**: This form captures information on patient impairment at paid and unpaid work due to breast cancer. This form is available in English (Appendix N) and Spanish (Appendix O) and patients should be allowed to use the language that they prefer.

**Timeline for completion**: prior to surgery, prior to radiation therapy, 6 months +/- 2 month, 12 months +/- 2 month, and 18 months +/- 2 month following completion of radiation.

### 4.5.13 EQ-5D-5L (v2.0) (Appendices Q & R)

**Purpose**: This form captures information on patient mobility, self care, usual activities, pain/discomfort, and anxiety/depression. This form is available in English (Appendix Q) and Spanish (Appendix R) and patients should be allowed to use the language that they prefer.

**Timeline for completion**: prior to surgery, prior to radiation therapy, 6 months +/- 2 month, 12 months +/- 2 month, and 18 months +/- 2 month following completion of radiation.

### 4.5.14. Cost Diary (Appendices S and T)

**Purpose:** These forms provide instructions to patients as well as a sample cost diary (Appendix S) and the cost diary (AppendixT) that will be provided to the patient. This diary captures information on the direct and indirect costs of medical care as assessed by the patient.

**Timeline for completion**: monthly from the end of radiation for 6 months during the duration of the study.

# 5.0 Statistics and Justification of Sample Size

## 5.1 Primary Endpoint

This is a study to investigate changes in and correlations between 1) lymphatic vessel anatomy and function, 2) peripheral blood/serum/plasma inflammatory cytokine levels, and 3) immune cell function, which may occur with development of lymphedema, characterized by arm swelling of at least 5% compared to baseline arm volume, after treatment for breast cancer. Based on observed changes in lymphatic pumping values in lymphedema study subjects in previous studies, we derived a sample size of 100 based on the following power analysis:

*Power Analysis:* This study plans to include 100 breast cancer subjects who will undergo ALND and radiation treatment. With 5% attrition per visit (23% overall attrition), leaving 77 study-finishing subjects, of whom published results suggest that 28-30% will develop arm LE, we will have 85% power with a two-sided significance level of 0.05 to detect a correlation of 0.6 or greater. To continually evaluate whether statistical significance can be ultimately achieved with reasonable numbers, we will follow the FDA Draft Guidance Document, “Adaptive Design Clinical Trials for Drugs and Biologics” to statistically assess the acquired data and continually power the study to adequately test the number of subjects required to achieve statistical significance.

For power calculation, we will compare each longitudinal continuous outcome (such as cytokine level, antibody level, etc.) between those who developed lymphedema and those who did not. Applying the formula for calculating power for longitudinal continuous outcome proposed by Diggle (2002) page 30, we obtain the minimum detectable difference between the two groups in unit of standard deviation of the outcome variable. Here we use the standard type I error rate=.05 and power=80%.^24^

| Sample Size LE Group | Sample Size No LE Group | Correlation among repeated observations | Minimum detectable unit of standard deviation |
| --- | --- | --- | --- |
| 22 | 55 | 0.1 | 0.40 |
|  |  | 0.2 | 0.45 |
|  |  | 0.4 | 0.52 |
|  |  | 0.6 | 0.59 |

From past studies NIRFLI we anticipate that 1 hour of imaging in several fields of view (dorsal hand, lateral and medial forearm, lateral and medial upper arm, axilla area, and breasts) will provide enough time for assessing the lymphatic “pumping” function by which statistical power calculations can be made. Values from NIRFLI and immune assay results of groups who do or do not develop LE within 18 months (matching BMIs where possible), as well as for single subjects longitudinally followed, will be compared and analyzed using Pearson’s or Spearman rank correlation, student’s t-test (for significance), ANOVA (for continuous variables, such as pulses per minute), and a linear mixed effects model (for pulsatile velocity). We will develop receiver operating characteristic (ROC) curves, using graduating values of one or more of the measured NIRFLI and immune parameters and increasing arm or breast volume measurements. Dr. Wenyaw Chan, Biostatistics Professor and Biostatistics Collaborator, at UTHSC-H, will advise on these statistical analysis and interpretation.

The following measures will be compared across lymphedema (arm volume change ≥ 5%) and no-lymphedema groups: 1) percent trunk swelling, 2) percent breast swelling, 3) percent extravascular dye, 4) lymphatic vessel tortuosity index, 5) lymphatic vessel dilation ratio, 6) plasma cytokine and chemokine levels, 7) plasma antinuclear antibody titers, 7) percent of certain T-cell populations in peripheral blood, 8) cytokine/chemokine levels in stimulated PBMC cultures, and 9) lymphatic vessel pumping frequency and velocity. For the purposes of comparing plasma cytokine levels, we will attempt to age match lymphedema and no-lymphedema subjects, because these values have been reported to vary with age. Comparisons of measures will be applied as indicated, using, for example, Bonferroni correction or false discovery rate.

We will attempt to derive Receiver Operator Curves (ROC analysis) for each measured parameter (i.e., percent extravascular dye, plasma cytokine level, vessel tortuosity index) compared to the “gold standard” of ≥ 5% arm volume change (specificity, sensitivity analysis).

## 5.2 Secondary Analyses

At each time point post surgery except the end of radiation, patients will be evaluated for lymphoedema via NIRFLI and via perometer. QuickDASH, EQ5D, LSIDS-A, patient lymphedema symptom survey instrument (PLSSI), work productivity acitivity impairment (WPAI) questionnaire, healthcare provider assesment of edema will also be measured longitudinally at the corresponding time points specified in the “Research Plans and Methods” section. These data will be summarized by time points using descriptive statistics such as frequency distribution, mean (± s.d.) and median (range) accompanied by graphical analysis^21^. Linear mixed effect models for the continuous measures such as LSIDS-A, and logistic regression models using generalized estimating equations (GEE) for the binary measures like healthcare provider assesment of arm edema^22^ (Liang, 1986) will be employed to take the intra-subject correlation into account to study the change of the measures over time and to compare the measures between patients with versus without lymphedema as diagnosed via NIRFLI and via perometer adjusting for other important covariates including patient demographic and disease characteristics. As a sensitivity analysis, we will classify patients into two groups, the group of patients who have never developed lymphedema vs the group of patients who developed lymphedema over the 18 months of follow-up. In patients who did not develop lymphedema, LSIDS-A as well the other secondary measures recorded at the earliest available of the post surgery, prior radiation, 6, 12 or 18 month visits will be used. In patients defined as cases of lymphedema, data from the earliest of these visits in which breast lymphedema is present, will be used. Addtionally, we will also compare the two groups using the worst and best value during follow-up for each of the measures.

For the optional costing analysis, a total of up to 50 patients will be included. We will apply cost-to-charge ratios to charges recorded in MDACC institutional databases and will obtain counts of various types of healthcare resources (e.g., physical therapy sessions, compressive bandages used to treat lymphedema). We will perform an assessment of the relative difference in time-driven activity based costing for the medical care delivered to breast cancer patients with versus without early onset lymphedema. Time-driven activity based costing will be performed to map the personnel and time resource utilization by breast cancer patients within the healthcare system. The cost per unit time of each provider with whom a patient comes into contact will be calculated, as will the quantity of time for each visit. For each patient, the time resource costs associated with breast cancer treatments and its related follow-up care will be collected. Means of utilization between those patients with and without early onset breast cancer-related lymphedema will be compared. For patients participating in the optional costing studies, the WPAI, EQ-5D, QuickDASH, and insurance cluster questions from the LSIDS-A questionnaires will be analyzed in this smaller cohort in the context of cost diary data.

# 6.0 Data Confidentiality Procedures

Since protected health information is obtained in this study, there is the possibility of the loss of confidentiality. To minimize this risk, enrollees will be assigned a code that will be used in lieu of personal identifiers wherever possible. Study records will be maintained at UTHSC-H in a secure location with controlled access (locked, restricted archives room with locked fireproof/waterproof file cabinets—room 330B in UTHSC-H’s Sarofim Research Building). Electronic study data will reside on a secure computer system equipped with a firewall and restricted access by username and password (UTHSC-H). All patient identifiers will be removed before data is disseminated in presentations and publications. As data is continually analyzed by UTHSC-H personnel, all de-identified analysis results will be promptly shared with MDACC research personnel using an institution-approved method (RedCAP).

Data will be stored indefinitely, because the near-infrared fluorescent images and immune assay results may be correlated to lymphedema parameters that are discovered later. Once the study is terminated, the data will be stored indefinitely at UTHSC-H in the locked archives room (paper) or the secure computer sites described above. The data may be used for future research, particularly the DNA genomic sequence data. Our R01 reviewers asked that we collect and use this data to find possible correlations between immune gene sequences and development of lymphedema, although the R01 budget will not cover this sequencing. We intend to apply for ancillary funding from NCI and other sources for the sequencing and analysis of the DNA genomic data. The consent form will allow subjects the option of sharing de-identified genomic DNA sequencing data with the National Institute of Health’s dbGap genomic database (future research). The consent form will also allow subjects the option of sharing aliquots of de-identified cells, plasma, and DNA with collaborating researchers who have access to equipment and expertise that are applicable to lymphedema research (future research--for example, epigenetic/methylation changes to DNA, although the cryopreserved cells and plasma will lose viability/robustness within 2-4 years of storage). The cells and plasma will be destroyed within 7 years of storage.

# 7.0 Informed Consent

Patients within the institution who are scheduled for definitive breast cancer treatment will be identified by either an attending physician in the Department of Radiation Oncology, Breast Surgery, or Breast Medical Oncology or by a research nurse or research data coordinator in the Department of Radiation Oncology. An informational flier (Appendix X) may be given to patients to provide them with information about the protocol. An informational flier (Appendix W) may be given to healthcare providers to provide them with information about the protocol.

# 8.0 References

1. McLaughlin, S.A. (2012). Lymphedema: separating fact from fiction. Oncology (Williston Park) 26, 242-9.
2. DeSantis, C.E., Lin, C.C., Mariotto, A.B., Siegel, R.L., Stein, K.D., Kramer, J.L., Alteri, R., Robbins, A.S., Jemal, A. (2014). Cancer treatment and survivorship statistics, 2014. CA Cancer J Clin 64, 252-71.
3. Foldi, M., Foldi, E. (2006). Foldi’s textbook of lymphology, 2^nd^ edition. Elsevier GmbH, Munich, Germany.
4. van Golen, R.F., van Gulik, T.M., Heger, M. (2012). The sterile immune response during hepatic ischemia/reperfusion. Cytokine Growth Factor Rev 23, 69-84.
5. Lee, M.J., Beith, J., Ward, L., Kilbreath, S. (2014). Lymphedema following taxane-based chemotherapy in women with early breast cancer. Lymphat Res Biol 12, 282-8.
6. Baker, A., Semple, J.L., Moore, S., Johnston, M. (2014). Lymphatic function is impaired following irradiation of a single lymph node. Lymphatic Res and Biol 12, 76-88.
7. Ohba, Y., Todo, Y., Kobayashi, N., Kaneuchi, M., Watari, H., Takeda, M., Sudo, S., Kudo, M., Kato, H., Sakuragi, N. (2011). Risk factors for lower-limb lymphedema after surgery for cervical cancer. Int J Clin Oncol 16, 238–243.
8. Shaitelman, S.F., Cromwell, K.D., Rasmussen, J.C., Stout, N.L., Armer, J.M., Lasinski, B.B., Cormier, J.N. (2015). Recent progress in the treatment and prevention of cancer-related lymphedema. CA Cancer J Clin 65, 55-81.
9. Chang, S.B., Askew, R.L., Xing, Y., Weaver, S., Gershenwald, J.E., Lee, J.E, Royal. R., Lucci, A., Ross, M.I., Cormier, J.N. (2010). Prospective assessment of postoperative complications and associated costs following inguinal lymph node dissection (ILND) in melanoma patients. Ann Surg Oncol 17, 2764–2772.
10. Armer, J.M., Stewart, B.R. (2010). Post-breast cancer lymphedema: incidence increases from 12 to 30 to 60 months. Lymphology 43, 118–127.
11. Avraham, T., Zampell, J.C., Yan, A., Elhadad, S., Weitman, E.S., Rockson, S.G., Bromberg, J., Mehrara, B.J. (2013). Th2 differentiation is necessary for soft tissue fibrosis and lymphatic dysfunction resulting from lymphedema. FASEB J 27, 1114-26.
12. Zampell, J.C., Yan, A., Elhadad, S., Avraham, T., Weitman, E., Mehrara, B.J. (2012). CD4^+^ cells regulate fibrosis and lymphangiogenesis in response to lymphatic fluid stasis. PLoS One 7, e49940.
13. Zampell, J.C., Yan, A., Avraham, T., Andrade, V., Malliaris, S., Aschen, S.Z., Rockson, S.G., Mehrara B.J. (2011). Temporal and spatial patterns of endogenous danger signal expression after wound healing and in response to lymphedema. Am J Physiol Cell Physiol 300, C1107-1121.
14. Leung, G., Baggott, C., West, C., Elboim, C., Paul, S.M., Cooper, B.A., Abrams, G., Dhruva, A., Schmidt, B.L., Kober, K., Merriman, J.D., Leutwyler, H., Neuhaus, J., Langford, D. Smoot, B.J., Aouizerat, B.E., Miaskowski, C. (2014). Cytokine candidate genes predict the development of secondary lymphedema following breast cancer surgery. Lymphat Res Biol 12, 1-13.
15. Aldrich, M.B., Guilliod, R., Fife, C.E., Maus, E.A., Smith, L., Rasmussen, J.C., Sevick-Muraca, E.M. (2012). Lymphatic abnormalities in the normal contralateral arms of subjects with breast cancer-related lymphedema as assessed by near-infrared fluorescent imaging. Biomed Opt Express 3, 1256-65.
16. Aldrich, M.B., Sevick-Muraca, E.M. (2013). Cytokines are systemic effectors of lymphatic function in inflammation. Cytokine 64, 362-9.
17. Joseph, W.J., Aschen, S., Ghanta, S., Cuzzone, D., Albano, N., Gardenier, J., Savetsky, I., Torrisi, J., Mehrera, B.J. (2014). Sterile inflammation after lymph node transfer improves lymphatic function and regeneration. Plast Reconstr Surg 134, 60-8.
18. Rasmussen, J.C., Tan, I-C., Marshall, M.V., Adams, K.E., Kwon, S., Fife, C.E., Maus, E.A., Smith, L.A., Covington, K.R., Sevick-Muraca, E.M. (2010). Human lymphatic architecture and dynamic transport imaged using near-infrared fluorescence. Transl Oncol 3, 362–372.
19. Marshall, M.V., Rasmussen, J.C., Tan, I.C., Aldrich, M.B., Adams, K.E., Wang, X., Fife, C.E., Maus, E.A., Smith, L.A., Sevick-Muraca, E.M. (2010). Near-infrared fluorescence imagingin humans with indocyanine green: a review and update. Open Surg Oncol J 2, 12-25.
20. Stout Gergich, N.L., Pfalzer, L.A., McGarvey, C., Springer, B., Gerber, L.H., Soballe, P. (2008). Preoperative assessment enables the early diagnosis and successful treatment of lymphedema. Cancer 112, 2809–19.

21. Woolson RaCW. Statistical Methods for the Analysis of Biomedical Data, 2nd Edition. New York: Wiley; 2002.

22. Liang KY, Zeger, S. L. Longitudinal data analysis using generalized linear models. Biometrika 1986;73:13-22.

23. Goosens MEJB, Rutten-van Molken MPMH, Vlaeyen JWS, van der Linden SMJP. (2000). The cost diary: a method to meaure direct and indirect costs in cost-effectiveness research. J Clin Epic 53, 688-695.

24. Diggle, P., Heagerty, P., Liang, K., and Zeger, S. (2002) Analysis of Longitudinal Data. 2^nd^ ed.Oxford.

**STROBE Checklist/Table**

| **Item and Number** | **Responses to recommendations or Locations within Manuscript** | **Page Number(s) or Location** |
| --- | --- | --- |
| 1. Title and abstract | a) “cohort, longitudinal, prospective” included in Abstract Methods  b) Abstract provides informative and balanced summary of what was done and findings | 1. 1 2. 2 |
| **Introduction** |  |  |
| 2) Background/rationale | manuscript Introduction | 3- |
| 3) Objectives | manuscript Introduction | Introduction, last paragraph |
| **Methods** |  |  |
| 4) Study Design | Manuscript Materials and Methods |  |
| 5) Setting | study location was MD Anderson Cancer Center, Houston, TX, USA, named in protocol in appendix, Abstract/Methods gives periods of recruitment and follow-up (2016-2021) | Manuscript pages 4 |
| 6) Participants | a) inclusion/exclusion criteria listed in study protocol provided in appendix, methods of participant selection described in Protocol in appendix, and follow-up methods described in manuscript Methods  b) not applicable—not a matched study | Appendix/Protocol page 3 and Manuscript page 4 |
| 7) Variables | Outcomes (statistically different pulsing frequency means amongst and between timepoints for ipsilateral ventral and ipsilateral axilla views, SARS-CoV-2 named as potential confounder, diagnostic criterion for BCRL given as relative volume change (RVC) >5% in Methods | Methods, Manuscript pages 5-7 |
| 8) Data sources/measurement | Pulsing frequency values determined by NIRF-LI | Manuscript pages 5-9 |
| 9) Bias | MBA, MM, JCR, and EMS-M independently assessed data collected, to avoid any bias in interpretation |  |
| 10) Study size | Power statistics performed at outset of study indicated that ~77 study finishers would suffice for most measurable outcomes (particularly lymphatic pulsing frequency, which we do not report in this manuscript). We began the study intending to consent 100 study subjects, and allowed for a 5% attrition rate per study visit (leaving ~77 finishers). Because SARS-CoV-2 slowed down/halted study recruitment, and the cohort consisted of advanced cancer patients, some of whom passed away due to cancer before completing the study, as well as some who dropped out, we consented 80 total. We prepared this manuscript using the 51 consented subjects who finished the study with at least 4 of the 5 imaging sessions completed. The results with this smaller study number of subjects were statistically significant. | Appendix/protocol pages 10-11 and Materials and Methods page 4 |
| 11) Quantitative variables | BCRL was defined as >5% arm swelling over baseline, pulses per minute were derived from NIRF-LI movies, using ImageJ freeware | Manuscript pages 4-5 |
| 12) Statistical methods | 1. Mixed-effects analysis calculated using standard methods. To control for confounding by SARS-CoV-2-driven study visit cancellation, 29 of the 80 enrolled study subjects were omitted from the analysis (fewer than 4 of the 5 scheduled imaging visits) 2. reasons for non-participation included death due to breast cancer before finishing the study, SARS-CoV-2 interruptions, and study subjects dropping out for individual reasons 3. participants missing study visits were not included in the pulsing frequency analysis 4. loss to follow-up was addressed by removing those study subjects who did not complete at least 4 of the 5 scheduled imaging visits 5. pairwise comparisons between any two timepoints were performed using t-tests of Least Squares Mean analysis | 1. Manuscript page 5-7 2. Manuscript page 5 3. Manuscript page 5 4. Manuscript page 5 5. Manuscript page 5 |
| **Results** |  |  |
| 13) Participants | a) 80 total subjects were eligible and consented—the 51-who completed at least 4 of the 5 scheduled imaging visits were included in the analysis of pulsing frequencies  b) of the enrolled 80, seven passed away before study completion, seven dropped out for individual reasons, two dropped out due to metastasis, and 13 missed multiple visits due to SARS-CoV-2 | a) Table 1  b) Table 1 |
| 14) Descriptive data | a) Table 1 lists demographic and clinical information for participants  b) no missing data for any participants in Table 1  c) scheduled follow-up times are described in Methods | a) Table 1  b) Table 1  c) Manuscript page 4 |
| 15) Outcome data | outcome events/measures are shown in Figures 3-5 | Figures 3-5 |
| 16) Main results | a) Mixed-effects analysis for comparisons of pulsatile frequency means at different timepoints are shown in Figure 5  b) ranges of continuous demographic variables are listed in Table 1, ranges of pulsing frequencies are shown in Figures 3, 5, and Supplementary Figures 1-3  c) relative risk/absolute risk not applicable | a) Figure 3  b) Table 1 and Figure 5  c) not applicable |
| 17) Other analyses | Not applicable | Not applicable |
| **Discussion** |  |  |
| 18) Key results | Key results are summarized with reference to study objectives in Discussion | Manuscript pages 5-9 |
| 19) Limitations | Limitations are discussed (Discussion) | Manuscript page 12 |
| 20) Interpretation | Interpretation given in Discussion and Conclusions | Manuscript pages 10-12 |
| 21) Generalizability | Conclusions state the generalizability of the results | Manuscript page 12 |
| Other information |  |  |
| 22) Funding | Funding sources identified in Acknowledgements | Manuscript page 12 |
